# Characterization of glycosphingolipids from gastrointestinal stromal tumours

**DOI:** 10.1038/s41598-020-76104-3

**Published:** 2020-11-09

**Authors:** Licínia Santos, Chunsheng Jin, Taťána Gazárková, Anders Thornell, Olov Norlén, Karin Säljö, Susann Teneberg

**Affiliations:** 1 Department of Medical Biochemistry and Cell Biology, Institute of Biomedicine, Sahlgrenska Academy, University of Gothenburg, P.O. Box 440, 405 30 Gothenburg, Sweden; 2 Department of Surgery, Institute of Clinical Sciences, Sahlgrenska Academy, University of Gothenburg, Gothenburg, Sweden; 3 Region Västra Götaland, Sahlgrenska University Hospital, Gothenburg, Sweden; 4 Department of Surgical Sciences, Uppsala University, Uppsala University Hospital, Uppsala, Sweden; 5 Department of Plastic Surgery, Institute of Clinical Sciences, Sahlgrenska Academy, University of Gothenburg, Gothenburg, Sweden

**Keywords:** Biochemistry, Cancer, Medical research

## Abstract

Gastrointestinal stromal tumours (GISTs) are the major nonepithelial neoplasms of the human gastrointestinal tract with a worldwide incidence between 11 and 15 per million cases annually. In this study the acid and non-acid glycosphingolipids of three GISTs were characterized using a combination of thin-layer chromatography, chemical staining, binding of carbohydrate recognizing ligands, and mass spectrometry. In the non-acid glycosphingolipid fractions of the tumors globotetraosylceramide, neolactotetraosylceramide, and glycosphingolipids with terminal blood group A, B, H, Le^x^, Le^a^, Le^y^ and Le^b^ determinants were found. The relative amounts of these non-acid compounds were different in the three tumour samples. The acid glycosphingolipid fractions had sulfatide, and the gangliosides GM3, GD3, GM1, Neu5Acα3neolactotetraosylceramide, GD1a, GT1b and GQ1b. In summary, we have characterized the glycosphingolipids of GISTs and found that the pattern differs in tumours from different individuals. This detailed characterization of glycosphingolipid composition of GISTs could contribute to recognition of new molecular targets for GIST treatment and sub-classification.

## Introduction

The most common mesenchymal neoplasms of the human gastrointestinal tract are gastrointestinal stromal tumours (GISTs). These tumours are rare, and the incidence is between 11 and 15 cases/million/year worldwide. The majority of the GISTs (60%) are located in the stomach, whereas 30% are in the jejunum or ileum, 5% in the duodenum and 4% in the colorectum^1,2^. GISTs arise from the interstitial cells of Cajal, also called the pacemaker cells for peristaltic contraction^1^. These cells are found in the muscularis propria and around the myenteric plexus along the gastrointestinal tract, and they have immunophenotypic and ultrastructural characteristics of both neural and smooth muscle elements^1^. Around 80% of GISTs have a mutation in the KIT tyrosine kinase gene, and just over 5% have a mutation in the KIT-homologous tyrosine kinase platelet-derived growth factor receptor alpha (PDGFRA) gene^3^. Hence, about 10% to 15% of GISTs are negative for KIT and PDGFRA mutations (termed “wild-type GISTs”)^4^. The type of mutation has prognostic importance and correlates with response to targeted drugs such as imatinib^3,5–7^.

Altered glycosylation is a common feature of cancer cells, and an increase in fucosylation, sialylation, and branching of glycans, is often found^8–10^. These changes may affect any type of glycoconjugate, such as *N*-glycans and *O*-glycans on glycoproteins, glycosphingolipids or proteoglycans. Certain glycan structures are well-known markers for tumour prognosis, and some serum tumour biomarkers presently used in clinical practice are serum glycoproteins, such as CEA, CA19-9, and CA-125 for colon, pancreatic, and ovarian cancer, respectively^11–13^. Thus, studies of aberrations in glycan structures can be used as targets to improve existing cancer biomarkers.

The present study is the first investigation of glycosphingolipids in GISTs. Acid and non-acid glycosphingolipids were isolated from three gastric tumours, and the glycosphingolipid fractions obtained were characterized by mass spectrometry and by binding of a battery of carbohydrate recognizing ligands. Sulfatide, and the gangliosides GM3, GD3, GM1, Neu5Acα3neolactotetraosylceramide, GD1a, GT1b and GQ1b were found in the acid glycosphingolipoid fractions. The non-acid glycosphingolipid fractions of the tumours had globotetraosylceramide, neolactotetraosylceramide, and glycosphingolipids with terminal blood group A, B, H, Le^x^, Le^a^, Le^y^ and Le^b^ determinants. The relative amounts of the non-acid compounds were different in the three tumour samples. This characterization of the glycosphingolipid patterns in GISTs might contribute to the development of new prognosis markers and therapeutic approaches.

## Results

### Isolation of glycosphingolipids

**Figure 1:**
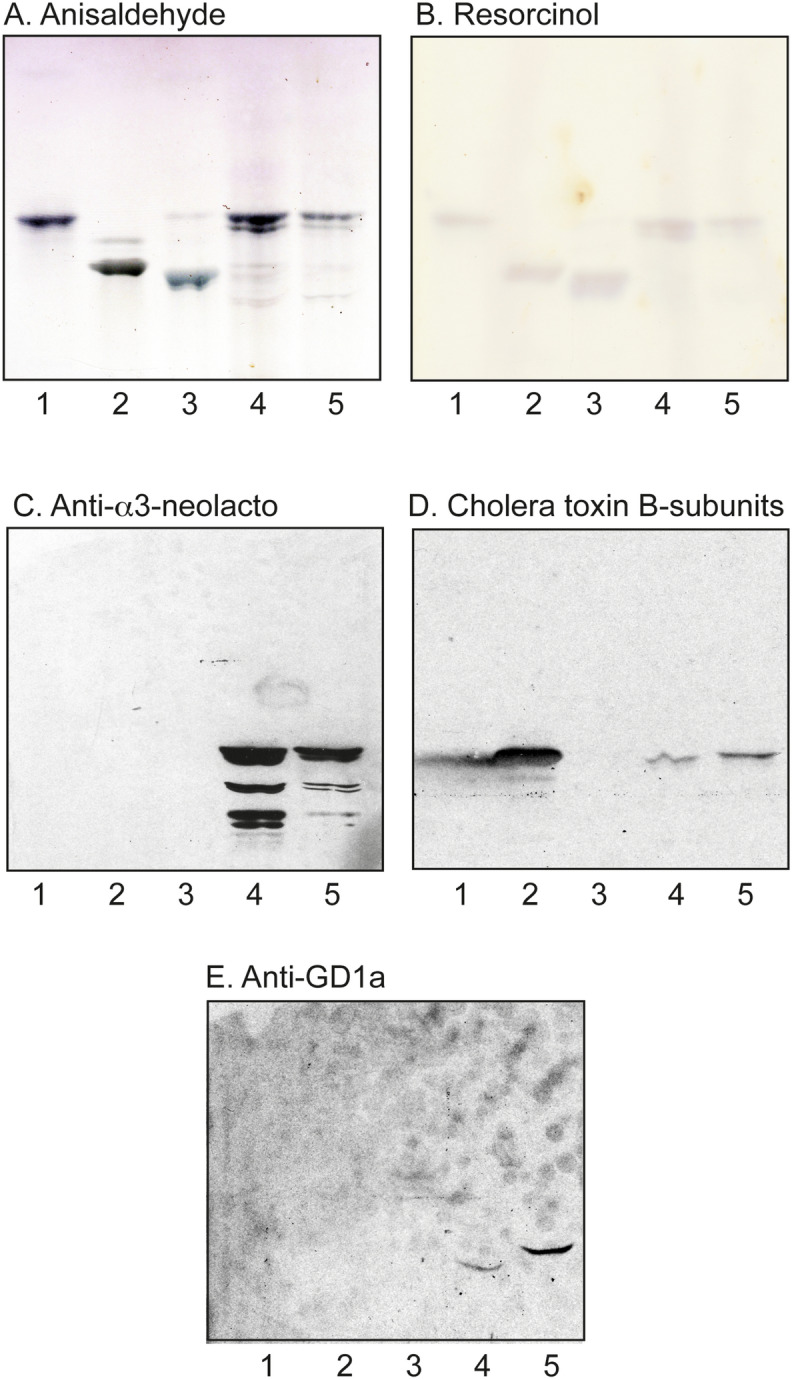
Thin-layer chromatography of the acid glycosphingolipids of GIST tumors, and binding of carbohydrate recognizing ligands. Thin-layer chromatogram after detection with anisaldehyde (**A**), and resorcinol (**B**), and autoradiograms obtained by binding of monoclonal antibodies directed against the Neu5Acα3GalβGlcNAc epitope (**C**), ganglioside GM1-recognizing cholera toxin B-subunits (**D**), and monoclonal antibodies directed against the ganglioside GD1a (**E**). The lanes were: lane 1, reference GM3 ganglioside, 4 µg; lane 2, reference GM1 ganglioside, 4 µg; lane 3, reference GD3 ganglioside, 4 µg; lane 4, total acid glycosphingolipids of GIST I, 40 µg; lane 5, total acid glycosphingolipids of GIST II, 40 µg.

Three gastric GIST tumours (denoted GIST I, GIST II and GIST III) were collected. GIST I was from a blood group A individual, GIST II from a blood group AB individual, and GIST III from a blood group O individual (Table 1). Total acid and non-acid glycosphingolipid fractions were isolated from the three tumours by standard procedures^14^. The amounts of acid and non-acid glycosphingolipids obtained are given in Table 1. The total acid fractions isolated from GIST I and II are shown in Fig. 1A,B, lanes 4 and 5. Both fractions had a major band co-migrating with reference GM3 ganglioside (lane 1), and also had a number of more slow-migrating bands.

**Table 1:** Amounts of acid and non-acid glycosphingolipids obtained from the GIST tumours.

	Weight (g)	Dry weight (g)	mg acid GSLs	mg acid GSLs/g dry weight	mg non-acid GSLs	mg non-acid GSLs/g dry weight
GIST I	22.0	4.4	19.0	4.3	5.0	1.2
GIST II	50.0	15.2	14.0	9.2	6.0	0.4
GIST III	8.4	1.1	2.9	2.6	4.0	3.6

**Figure 2:**
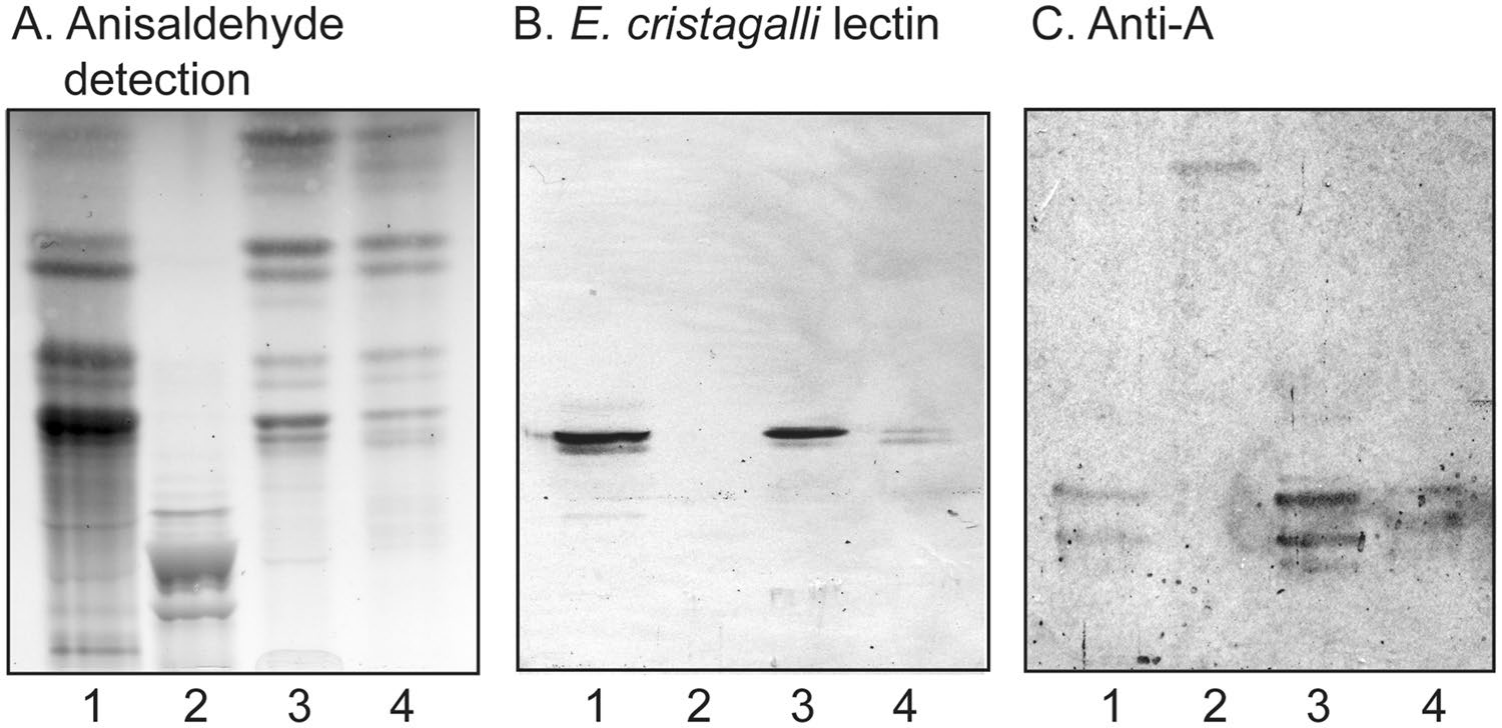
Thin-layer chromatography of the non-acid glycosphingolipids of GIST tumors, and binding of carbohydrate recognizing ligands. Thin-layer chromatogram after detection with anisaldehyde (**A**), and autoradiograms obtained by binding of, the Galβ4GlcNAc-/Fucα2Galβ4GlcNAc-binding lectin from *E. cristagalli* (**B**), and monoclonal antibodies directed against the blood group A determinant (**C**). The lanes were: lane 1, reference total non-acid glycosphingolipids of human blood group AB erythrocytes, 40 µg: lane 2, reference calf brain gangliosides, 40 µg: lane 3, total non-acid glycosphingolipids of GIST I, 40 µg; lane 4, total non-acid glycosphingolipids of GIST II, 40 µg.

The total non-acid glycosphingolipid fractions had a number of compounds migrating as mono-, di-, tri and tetraglycosylceramides, and also some minor slow-migrating compounds (exemplified by GIST I and II in Fig. 2A, lanes 3 and 4).

### Characterization of total acid GIST glycosphingolipids

#### LC-ESI/MS

**Figure 3:**
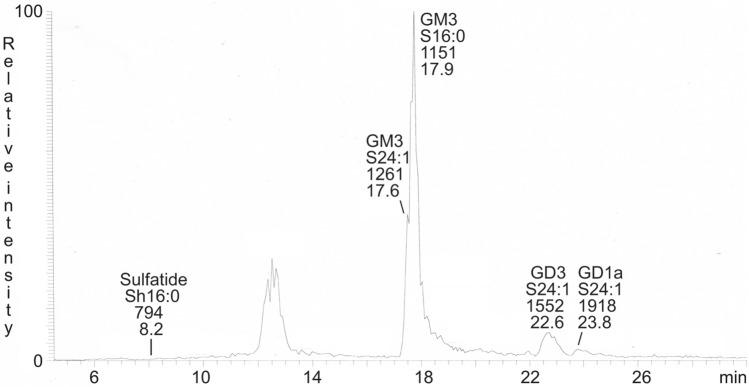
Base peak chromatogram from LC-ESI/MS of the total acid glycosphingolipid fraction from GIST II. The identification of glycosphingolipids was based on their retention times, determined molecular masses and subsequent MS^2^ sequencing. The data were processed using the Xcalibur software (version 2.0.7, Thermo Scientific, www.thermofisher.com). The glycosphingolipids identified in the chromatograms were: Sulfatide, SO_3_-3Galβ1Cer; Neu5Ac-GM3, Neu5Acα3Galβ4Glcβ1Cer; Neu5Ac-GD3, Neu5Acα8Neu5Acα3Galβ4Glcβ1Cer; Neu5Ac-GD1a, Neu5Acα3Galβ3GalNAcβ4(Neu5Acα3)Galβ4Glcβ1Cer. In the shorthand nomenclature for fatty acids and bases, the number before the colon refers to the carbon chain length and the number after the colon gives the total number of double bonds in the molecule. Fatty acids with a 2-hydroxy group are denoted by the prefix h before the abbreviation *e.g*. h16:0. For long chain bases, S designates sphingosine (d18:1).

The native total acid glycosphingolipids fractions from GIST I and II were analyzed by LC-ESI/MS (exemplified in Fig. 3). In both cases the major molecular ions were from the GM3 ganglioside with d18:1–16:0 ceramide (*m/z* 1151) and d18:1–24:1 ceramide (*m/z* 1261). In the case of GIST II there were also molecular ions of the GD3 ganglioside with d18:1–24:1 ceramide (*m/z* 776) and the GD1a ganglioside with d18:1–24:1 ceramide (*m/z* 959). A minor ion at *m/z* 794 was found by a search for molecular ions of sulfatide, and MS^2^ of this ion gave a characteristic sulfatide spectrum (Supplemental Figure S1)^15^.

#### Chromatogram binding assays

The binding of a number of carbohydrate binding ligands to the total acid glycosphingolipids fractions from GIST I and II were next examined (Fig. 1). Here the monoclonal antibodies directed against the Neu5Acα3Galβ4GlcNAc sequence recognized several slow-migrating compounds in the acid fractions from the two GISTs (Fig. 1C, lanes 4 and 5), indicating the presence of complex gangliosides with neolacto (Galβ4GlcNAc) core chains. Presence of the GM1 ganglioside was indicated by the binding of cholera toxin B-subunits (Fig. 1D, lanes 4 and 5). The monoclonal antibodies directed against the GD1a ganglioside also bound to the acid fractions from the two GIST tumours (Fig. 1E, lanes 4 and 5), whereas there was no binding of anti-GD3 antibodies or antibodies directed against the Neu5Acα6Galβ4GlcNAc sequence (not shown).

### Separation of the acid glycosphingolipids of GIST II

**Figure 4:**
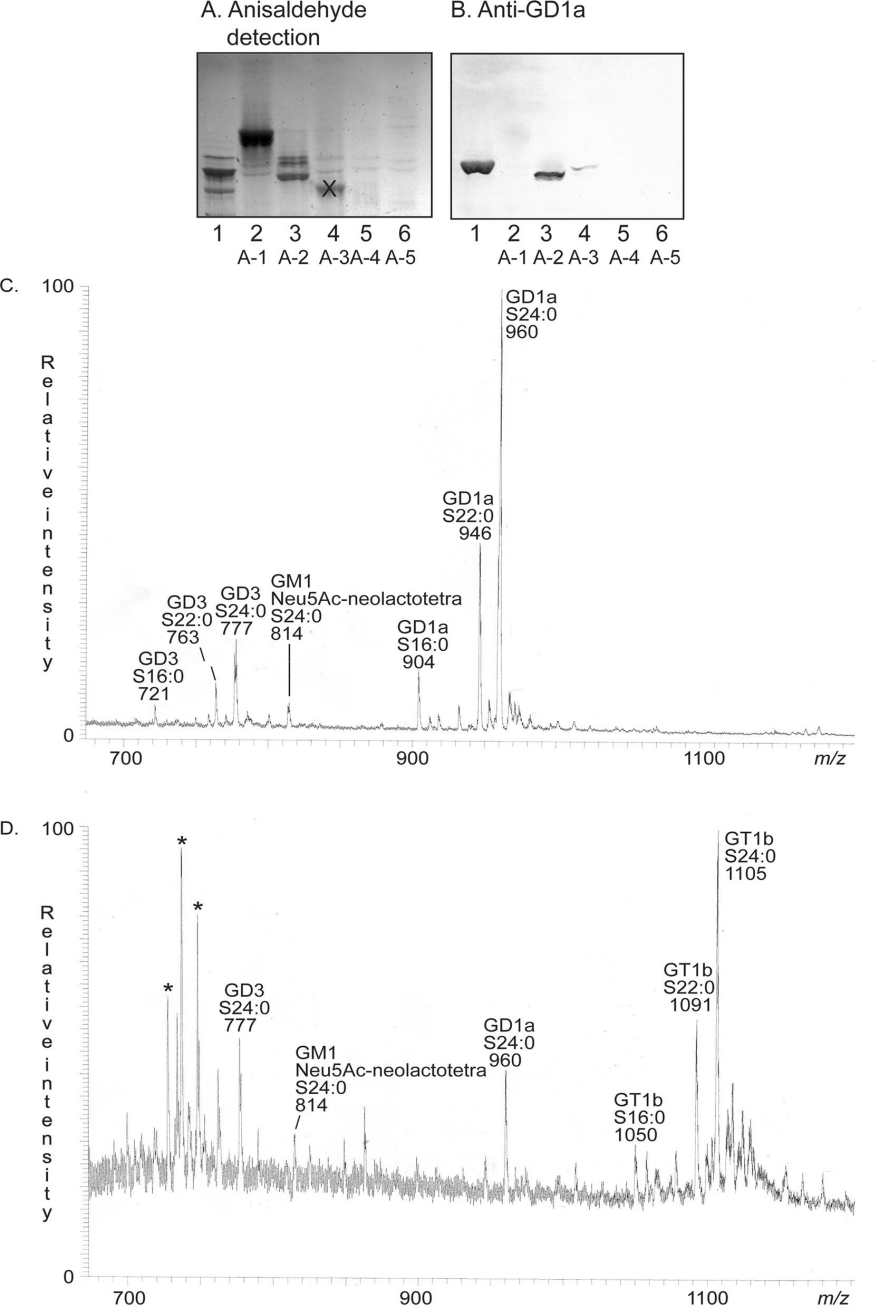
Characterization of the acid glycosphingolipid subfractions isolated from GIST II. Thin-layer chromatogram after detection with anisaldehyde (**A**), and autoradiogram obtained by binding of monoclonal antibodies directed against the ganglioside GD1a (**B**). The lanes were: lane 1, reference calf brain gangliosides, 40 µg; lanes 2–6, acid GIST fractions A-1–A-5, 20 µg/lane. (**C**) Molecular ion profile from LC-ESI/MS of fraction A-2. (**D**) Molecular ion profile from LC-ESI/MS of fraction A-4. The identification of glycosphingolipids was based on their retention times, determined molecular masses and subsequent MS^2^ sequencing. The data were processed using the Xcalibur software (version 2.0.7, Thermo Scientific, www.thermofisher.com). In the shorthand nomenclature for fatty acids and bases, the number before the colon refers to the carbon chain length and the number after the colon gives the total number of double bonds in the molecule. For long chain bases, S designates sphingosine (d18:1). * marks non-glycosphingolipid contaminants.

Since very little information was obtained from these LC-ESI/MS analyses the total acid glycosphingolipid fraction of GIST II was further separated on an Iatrobeads column eluted with increasing volumes of methanol in chloroform. This gave one fraction containing slow-migrating compounds (18 mg), and this fraction was further separated into subfractions that were pooled according to their mobility on thin-layer chromatograms. This gave five subfractions which were denoted fractions A-1 to A-5 (Fig. 4A, lanes 2–6).

### LC-ESI/MS of fractions A1–A5

LC-ESI/MS of fraction A-1 gave two major molecular ions at *m/z* 1151 and *m/z* 1263. MS^2^ of these ions identified the GM3 ganglioside with d18:1–16:0 and d18:1–24:0 ceramides, respectively (data not shown).

A number of doubly charged ([M − 2H^+^]^2−^) molecular ions were obtained by LC-ESI/MS of fraction A-2. MS^2^ identified the major molecular ion as the GD1a ganglioside with d18:1–16:0 ceramide (*m/z* 903), d18:1–22:0 ceramide (*m/z* 946), and d18:1–24:0 ceramide (*m/z* 960) (Fig. 4C). The presence of the GD1a ganglioside in fraction A-2 was in line with the binding of anti-GD1a antibodies to this fraction (Fig. 4B, lane 3).

There were also [M − 2H^+^]^2−^ ions of the GD3 ganglioside (*m/z* 721, *m/z* 763 and *m/z* 777), and of Neu5Ac-neolactotetraosylceramide or the GM1 ganglioside (*m/z* 814).

The base peak chromatogram obtained from fraction A-3 was weak and only had one [M − 2H^+^]^2−^ ion at *m/z* 777 corresponding to the GD3 ganglioside and one [M − 2H^+^]^2−^ ion at *m/z* 960 corresponding to the GD1a ganglioside, both with d18:1–24:0 ceramide (data not shown).

**Figure 5:**
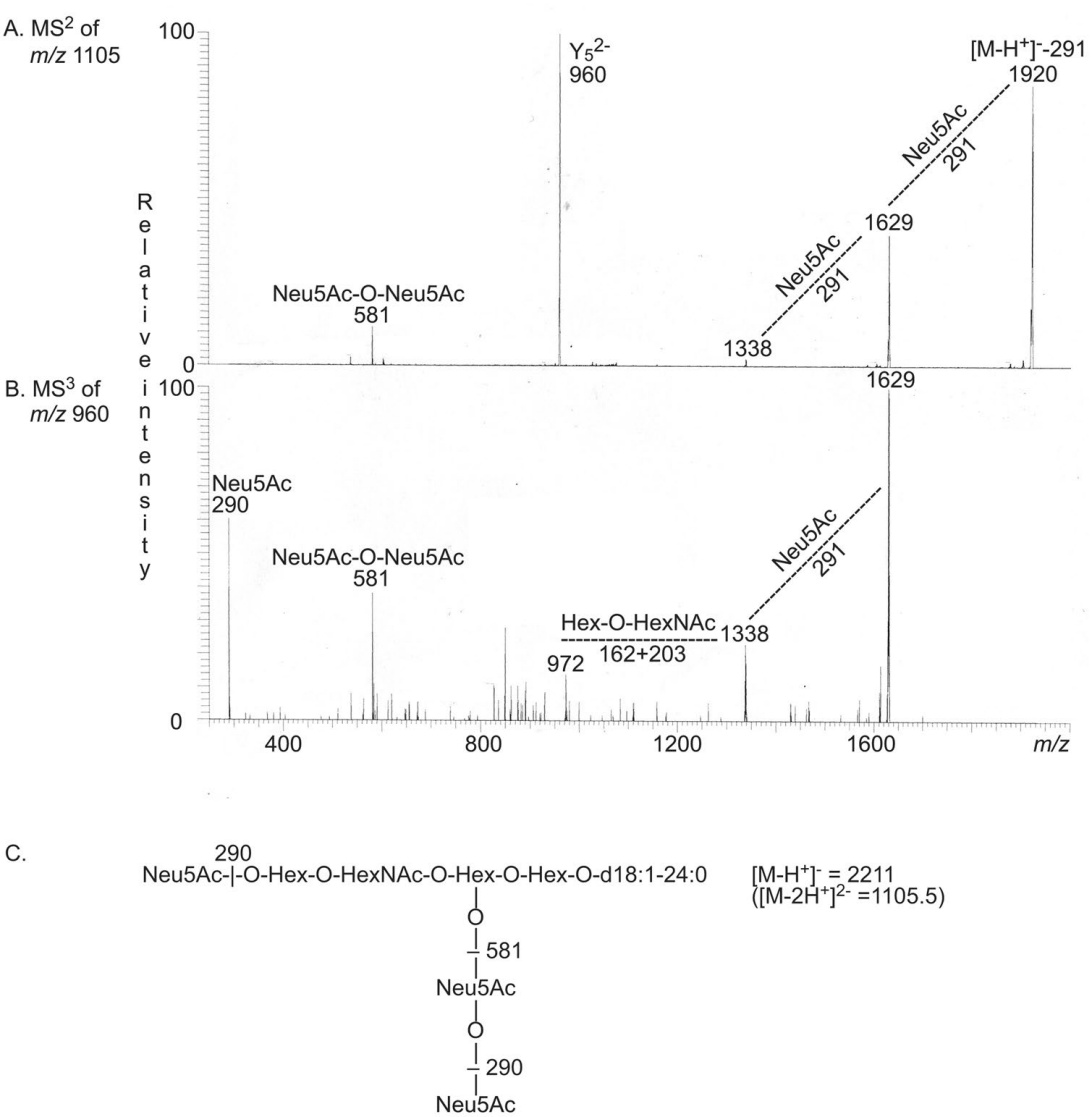
LC-ESI/MS of fraction A-4. (**A**) MS^2^ of the [M − 2H^+^]^2−^ ion at *m/z* 1105. (**B**) MS^3^ of the ion at *m/z* 960. (**C**) The interpretation formula shows the deduced ganglioside structure. The data were processed using the Xcalibur software (version 2.0.7, Thermo Scientific, www.thermofisher.com).

[M − 2H^+^]^2−^ ions at *m/z* 777 and at *m/z* 960 corresponding to the GD3 and GD1a gangliosides were also found by LC-ESI/MS of fraction A-4 (Fig. 4D). Here the major compound gave a [M − 2H^+^]^2−^ ion at *m/z* 1105.5. MS^2^ of *m/z* 1105.5 demonstrated a ganglioside with three Neu5Ac, three Hex and one HexNAc and d18:1–24:0 ceramide (Fig. 5A). The ion at *m/z* 581 demonstrated a Neu5Ac-Neu5Ac sequence, while the ions at *m/z* 1920, *m/z* 1629 and *m/z* 1338 were due to loss of one, two and three Neu5Ac from the molecular ion. MS^3^ of *m/z* 960 also gave ions at *m/z* 1629 and *m/z* 1338 caused by loss of one and two Neu5Ac, and also an ion at m/z 972 due to loss of a Hex and a HexNAc (Fig. 5B). Taken together this demonstrated a GT1b ganglioside with d18:1–24:0 ceramide (Fig. 5C). In the same manner the GT1b ganglioside with d18:1–16:0 ceramide was identified by MS^2^ and MS^3^ of the [M − 2H^+^]^2−^ ion at *m/z* 1049.5, and the GT1b ganglioside with d18:1–22:0 ceramide by MS^2^ and MS^3^ of the [M − 2H^+^]^2−^ ion at *m/z* 1091.

**Figure 6:**
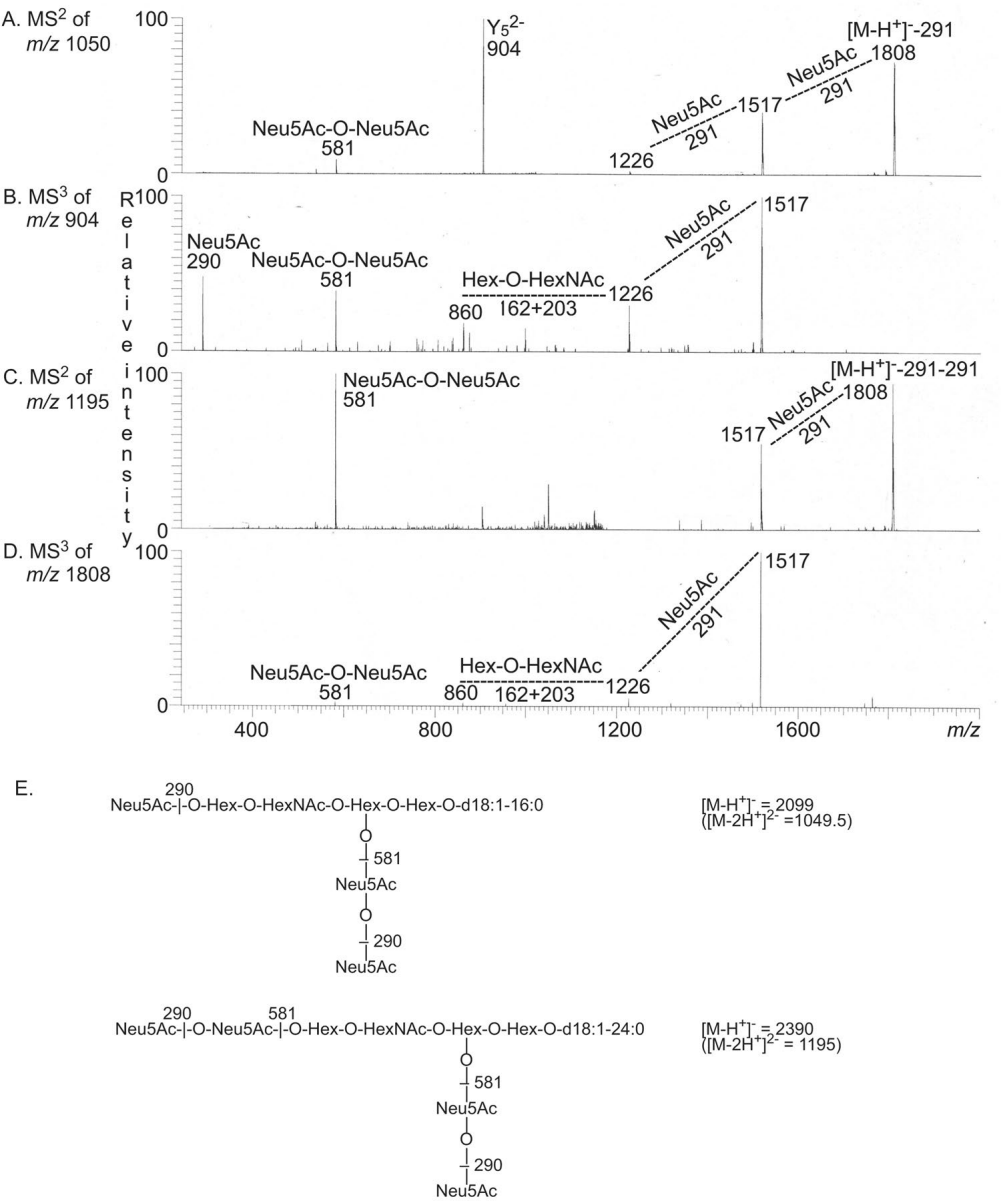
LC-ESI/MS of fraction A-5. (**A**) MS^2^ of the [M − 2H^+^]^2−^ ion at *m/z* 1050. (**B**) MS^3^ of the ion at *m/z* 904. (**C**) MS^2^ of the [M − 2H^+^]^2−^ ion at *m/z* 1195. (**D**) MS^3^ of the ion at *m/z* 1808. (**C**) The interpretation formulas show the deduced ganglioside structures. The data were processed using the Xcalibur software (version 2.0.7, Thermo Scientific, www.thermofisher.com).

LC-ESI/MS of fraction A-5 gave a major [M − 2H^+^]^2−^ ion at *m/z* 1049.5, and the GT1b ganglioside with d18:1–16:0 ceramide was again identified by MS^2^ and MS^3^ of this ion (Fig. 6A,B,E). The base peak chromatogram also had a [M − 2H^+^]^2−^ ion at *m/z* 1195 ([M − H^+^]^−^ ion at *m/z* 2390) corresponding to a ganglioside with four Neu5Ac, three Hex and one HexNAc and d18:1–24:0 ceramide. By MS^2^ and MS^3^ of the ion at *m/z* 1195 the GQ1b ganglioside with d18:1–24:0 ceramide was tentatively characterized (Fig. 6C,D,E).

The gangliosides characterized in in the acid glycosphingolipid fraction of GIST II are summarized in Table 2.

**Table 2:** Gangliosides of GIST II identified by LC-ESI/MS.

Trivial name	Structure
GM3	Neu5Acα3Galβ4Glcβ1Cer
GM1	Galβ3GalNAcβ4(Neu5Acα3)Galβ4Glcβ1Cer
Neu5Acα3neolacto4	Neu5Acα3Galβ4GlcNAcβ3Galβ4Glcβ1Cer
GD3	Neu5Acα8Neu5Acα3Galβ4Glcβ1Cer
GD1a	Neu5Acα3Galβ3GalNAcβ4(Neu5Acα3)Galβ4Glcβ1Cer
GT1b	Neu5Acα3Galβ3GalNAcβ4(Neu5Acα8Neu5Acα3)Galβ4Glcβ1Cer
GQ1b	Neu5Acα8Neu5Acα3Galβ3GalNAcβ4(Neu5Acα8Neu5Acα3)Galβ4Glcβ1Cer

### Characterization of total non-acid GIST glycosphingolipids

#### LC-ESI/MS of native glycosphingolipids

**Figure 7:**
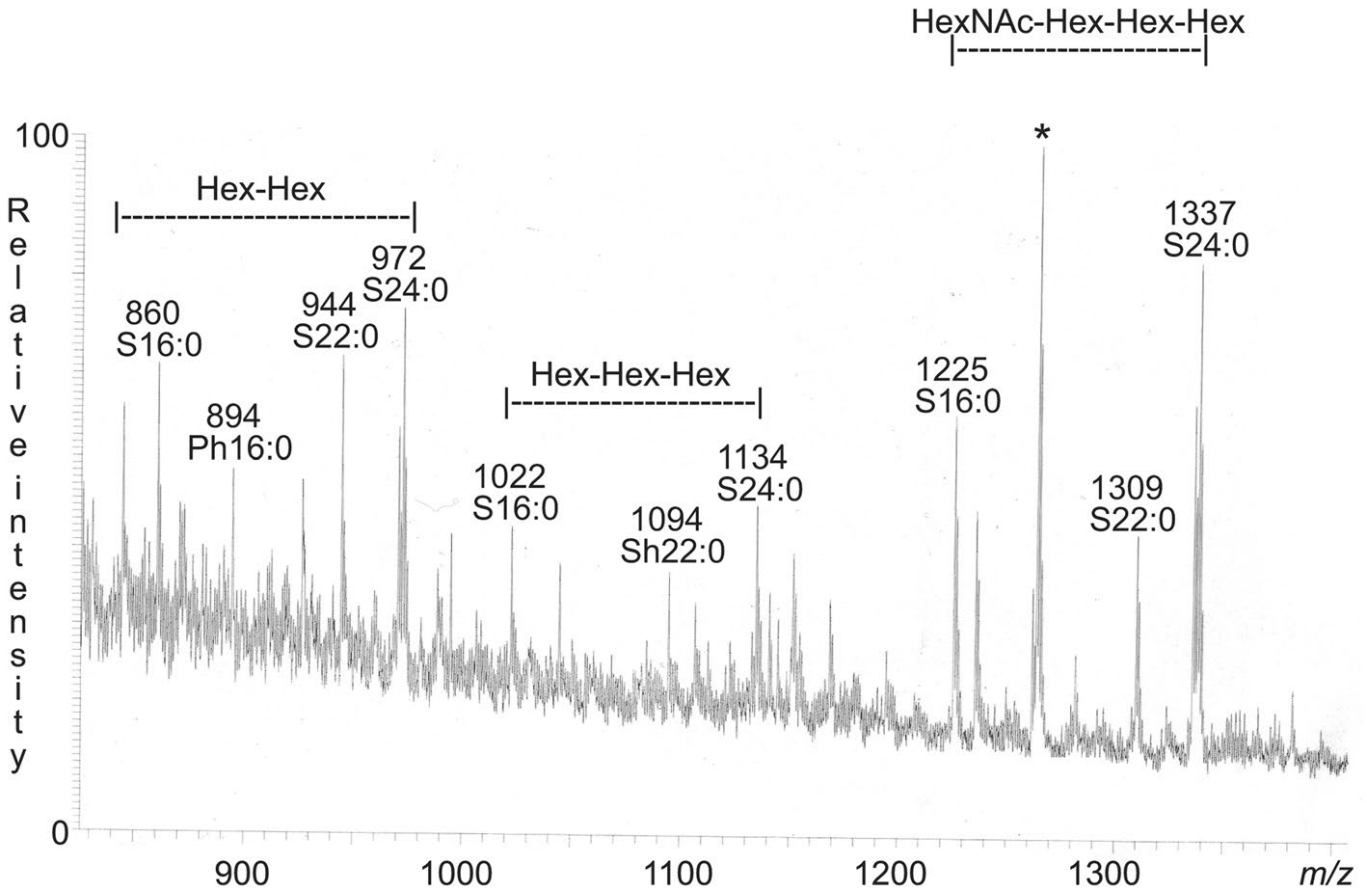
Molecular ion profile from LC-ESI/MS of the total non-acid glycosphingolipid fraction from GIST II. The identification of glycosphingolipids was based on their retention times, determined molecular masses and subsequent MS^2^ sequencing. The data were processed using the Xcalibur software (version 2.0.7, Thermo Scientific, www.thermofisher.com). In the shorthand nomenclature for fatty acids and bases, the number before the colon refers to the carbon chain length and the number after the colon gives the total number of double bonds in the molecule. Fatty acids with a 2-hydroxy group are denoted by the prefix h before the abbreviation *e.g*. h16:0. For long chain bases, S designates sphingosine (d18:1) and P phytosphingosine (t18:0). *marks a non-ganglioside contaminant.

To get an overview of the ceramide composition the native non-acid glycosphingolipid fractions of GIST I and GIST II were first analyzed by LC-ESI/MS using a polyamine column (exemplified in Fig. 7). Thereby dihexosylceramide, trihexosylceramide, and a tetraosylceramide with one HexNAc and three Hex, all with mainly sphingosine and non-hydroxy fatty acids with 16 and 24 carbon atoms were identified.

#### LC-ESI/MS of glycosphingolipid-derived oligosaccharides

Thereafter the non-acid glycosphingolipid fractions were hydrolyzed with endoglycoceramidase II from *Rhodococcus sp*., and the oligosaccharides thereby obtained were characterized by LC-ESI/MS using a graphitized carbon column. By LC-ESI/MS of oligosaccharides on graphitized carbon columns a resolution of isomeric oligosaccharides is obtained, and by MS^2^ a series of C-type ions is obtained which gives the carbohydrate sequence^16^. Furthermore, the MS^2^ spectra of oligosaccharides with a Hex or HexNAc substituted at C-4 have diagnostic cross-ring^0,2^A-type fragment ions which allow identification of linkage positions^16,17^. Thus, MS^2^ spectra of oligosaccharides with globo (Galα4Gal) or type 2 (Galβ4GlcNAc) core structures have such ^0,2^A-type fragment ions, but not the spectra obtained from oligosaccharides with isoglobo (Galα3Gal) or type 1 (Galβ3GlcNAc) core chains.

**Figure 8:**
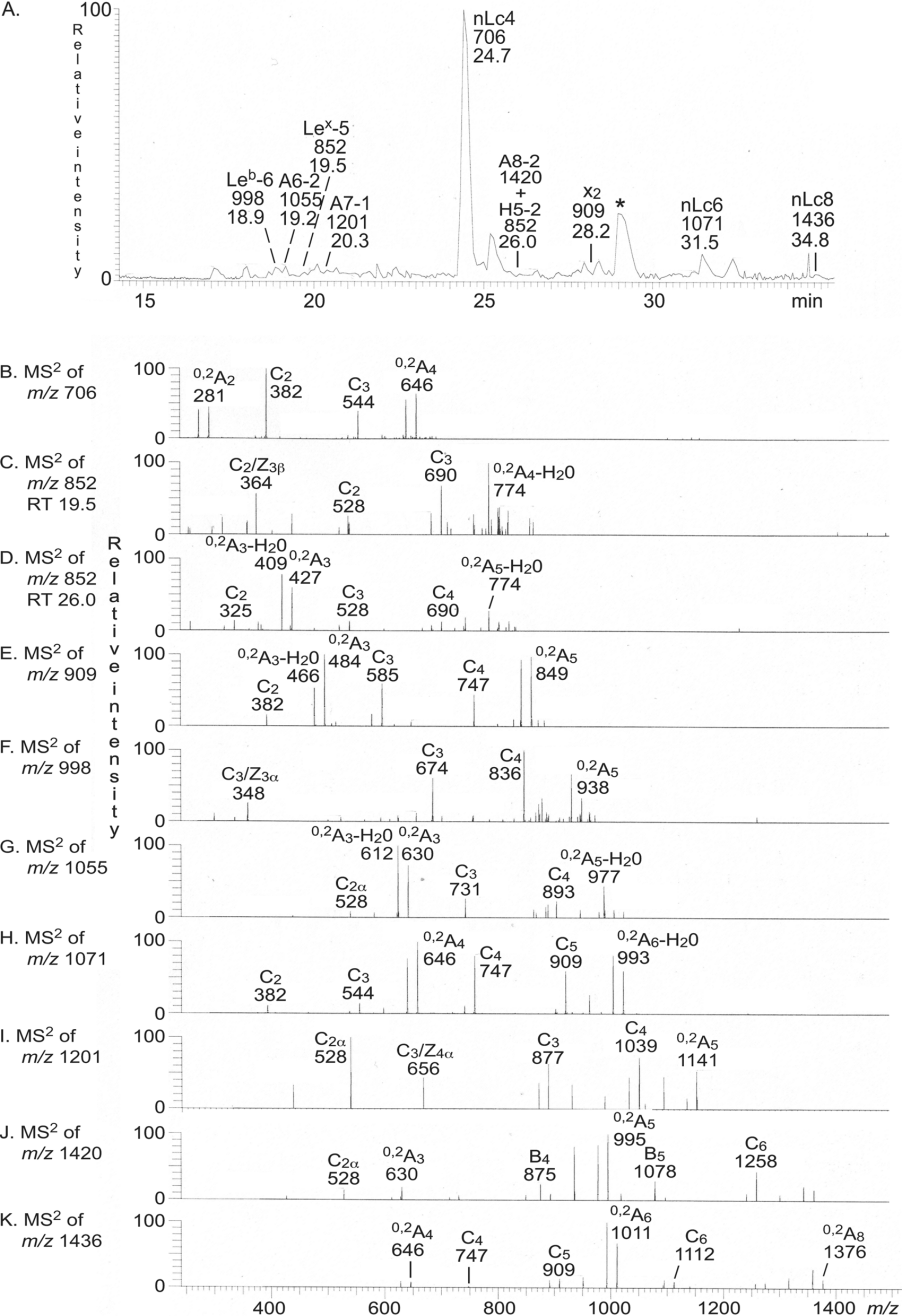
Characterization of the non-acid glycosphingolipids of GIST I. (**A**) Base peak chromatogram from LC-ESI/MS of the oligosaccharides obtained from the total non-acid glycosphingolipid fractions from GIST I by hydrolysis with endoglycoceramidase II from *Rhodococcus* spp. (**B**) MS^2^ of the [M − H^+^]^−^ ion at *m/z* 706 (retention time 24.7 min). (**C**) MS^2^ of the [M − H^+^]^−^ ion at *m/z* 852 (retention time 19.2 min). (**D**) MS^2^ of the [M − H^+^]^−^ ion at *m/z* 852 (retention time 26.0 min). (**E**) MS^2^ of the [M − H^+^]^−^ ion at *m/z* 909 (retention time 28.3 min). (**F**) MS^2^ of the [M − H^+^]^−^ ion at *m/z* 998 (retention time 19.0 min). (**G**) MS^2^ of the [M − H^+^]^−^ ion at *m/z* 1055 (retention time 19.2 min). (**H**) MS^2^ of the [M − H^+^]^−^ ion at *m/z* 1071 (retention time 31.7 min). (**I**) MS^2^ of the [M − H^+^]^−^ ion at *m/z* 1201 (retention time 20.3 min). (**J**) MS^2^ of the [M − H^+^]^−^ ion at *m/z* 1420 (retention time 26.0 min). (**K**) MS^2^ of the [M − H^+^]^−^ ion at *m/z* 1436 (retention time 34.8 min). See Supplementary Figure S2 for interpretation formulas. The data were processed using the Xcalibur software (version 2.0.7, Thermo Scientific, www.thermofisher.com). The oligosaccharides identified in the chromatograms were: Le^b^-6, Fucα2Galβ3(Fucα4)GlcNAcβ3Galβ4Glc; A6-2, GalNAcα3(Fucα2)Galβ4GlcNAcβ3Galβ4Glc; Le^x^-5, Galβ4(Fucα3)GlcNAcβ3Galβ4Glc; A7-1, GalNAcα3(Fucα2)Galβ3(Fucα4)GlcNAcβ3Galβ4Glc; nLc4, Galβ4GlcNAcβ3Galβ4Glc; A8-2, GalNAcα3(Fucα2)Galβ4GlcNAcβ3Galβ4GlcNAcβ3Galβ4Glc H5-2, Fucα2Galβ4GlcNAcβ3Galβ4Glc; x_2_, GalNAcβ3Galβ4GlcNAcβ3Galβ4Glc; nLc6,Galβ4GlcNAcβ3Galβ4GlcNAcβ3Galβ4Glc; nLc8, Galβ4GlcNAcβ3Galβ4GlcNAcβ3Galβ4GlcNAcβ3Galβ4Glc. *marks a non-oligosaccharide contaminant.

The base peak chromatogram from LC-ESI/MS of the oligosaccharides obtained from GIST I (Fig. 8A) had a number of molecular ions corresponding to tetrasaccharides (detected as [M − H^+^]^−^ ions at *m/z* 706) to heptasaccharides (detected as [M − H^+^]^−^ ions at *m/z* 1201). The predominant molecular ion was found at *m/z* 706 at retention time 24.7 min. These molecular ions were subjected to MS^2^ (Fig. 8 and Supplementary Fig. S2).

A prominent ^0,2^A_2_ fragment ion at *m/z* 281 demonstrating a terminal Hex-HexNAc sequence with a 4-substituted HexNAc, *i.e*. a type 2 core chain (Galβ4GlcNAc) was present in the MS^2^ spectrum of the ion at *m/z* 706 (Fig. 8B). There was also a C_2_ ion at *m/z* 382 and a C_3_ ion at *m/z* 544, and taken together this demonstrated a neolacto tetrasaccharide (Galβ4GlcNAcβ3Galβ4Glc).

The MS^2^ spectrum of the ion at *m/z* 852 at retention time 19.5 min (Fig. 8C) had a fragment ion at *m/z* 364. This type of fragment ion is caused by a double glycosidic cleavage of the 3-linked branch at C_3_ and Z_3_β, and is diagnostic for an internal 4-linked GlcNAc with a Fuc at 3-position^17^. The MS^2^ spectrum also had a C_2_ ion at *m/z* 528 and a C_3_ ion at *m/z* 690. Taken together these spectral features identified a Le^x^ pentasaccharide (Galβ4(Fucα3)GlcNAcβ3Galβ4Glc).

MS^2^ of the molecular ion at *m/z* 852 eluting at 26.0 min (Fig. 8D) identified a Fuc-Hex-HexNAc-Hex-Hex pentasaccharide. This was conluded from the series of C-type fragment ions (C_2_ at *m/z* 325, C_3_ at *m/z* 528, and C_4_ at *m/z* 690. 4-substitution of the HexNAc, *i.e*. a type 2 core chain (Galβ4GlcNAc) was indicated by the ^0,2^A_3_-H_2_O fragment ion at *m/z* 409 and the ^0,2^A_3_ fragment ion at *m/z* 427. Taken together this identified a H type 2 pentasaccharide (Fucα2Galβ4GlcNAcβ3Galβ4Glc).

A pentasaccharide with HexNAc-Hex-HexNAc-Hex-Hex sequence was identified by the C-type fragment ion series (C_2_ at *m/z* 382, C_3_ at *m/z* 585, and C_4_ at *m/z* 747) obtained by MS^2^ of the molecular ion at *m/z* 909 (Fig. 8E). Here the ^0,2^A_3_ fragment ion at *m/z* 484, and the ^0,2^A_3_-H_2_O ion at *m/z* 466, demonstrated a 4-substituted internal HexNAc. These features of the MS^2^ spectrum allowed identification of an x_2_ pentasaccharide (GalNAcβ3Galβ4GlcNAcβ3Galβ4Glc).

The MS^2^ spectrum obtained from the ion at *m/z* 998 (Fig. 8F) had a prominent fragment ion at *m/z* 348. This type of ion is diagnostic for an internal 3-linked GlcNAc substituted with a Fuc at C-4^17^, and is a double glycosidic cleavage of the 3-linked branch at C_3_ and Z_3_α. C-type fragment ions were present at *m/z* 674 (C_3_) and *m/z* 836 (C_4_), and taken together this indicated a Le^b^ hexasaccharide (Fucα2Galβ3(Fucα4)GlcNAcβ3Galβ4Glc).

The series of C-type fragment ions (C_2_ at *m/z* 528, C_3_ at *m/z* 731, and C_4_ at *m/z* 893) from MS^2^ of the ion at *m/z* 1055 (Fig. 8G) demonstrated a HexNAc-(Fuc)Hex-HexNAc-Hex-Hex sequence. A type 2 core chain (Galβ4GlcNAc), *i.e*. substitution of the internal HexNAc at C4, was demonstrated by the ^0,2^A_3_ fragment ion at *m/z* 630, and the ^0,2^A_4_-H_2_O fragment ion at *m/z* 612. Thus, a blood group A type 2 hexasaccharide (GalNAcα3(Fucα2)Galβ4GlcNAcβ3Galβ4Glc) was identified.

A neolacto hexasaccharide (Galβ4GlcNAcβ3Galβ4GlcNAcβ3Galβ4Glc) was characterized by MS^2^ of the ion at *m/z* 1071 (Fig. 8H). This was deduced from the C-type fragment ion series (C_2_ at *m/z* 382, C_3_ at *m/z* 544, C_4_ at *m/z* 747, and C_5_ at *m/z* 909), demonstrating a Hex-HexNAc-Hex-HexNAc-Hex-Hex carbohydrate sequence, along with the ^0,2^A_4_ fragment ion at *m/z* 646, which demonstrated 4-substitution of the innermost HexNAc.

MS^2^ of the molecular ion at *m/z* 1201 (Fig. 8I) gave a spectrum that was very similar to the MS^2^ spectrum obtained from reference blood group type 1/ALe^b^ heptasaccharide^18^. The series of C-type fragment ions (C_2_ at *m/z* 528, C_3_ at *m/z* 877, and C_4_ at *m/z* 1039) demonstrated a HexNAc-(Fuc-)Hex-(Fuc-)HexNAc-Hex-Hex sequence. The spectrum also had an ion at *m/z* 656, caused by a double glycosidic cleavage of the 3-linked branch at C_3_ and Z_4_α^17^, and thus signifying an internal 3-linked GlcNAc substituted with a Fuc at C-4. Thus, this demonstrated a blood group A type 1/ALe^b^ heptasaccharide (GalNAcα3(Fucα2)Galβ3(Fucα4)GlcNAcβ3Galβ4Glc).

The series of B-type and C-type fragment ions (C_2_ at *m/z* 528, B_4_ at *m/z* 875, B_5_ at *m/z* 1078, and C_6_ at *m/z* 1258) obtained by MS^2^ of the ion at *m/z* 1420 (Fig. 8J) indicated a HexNAc-(Fuc)Hex-HexNAc-Hex-HexNAc-Hex-Hex sequence. The ^0,2^A_3_ fragment ion at *m/z* 630, and the ^0,2^A_5_ fragment ion at *m/z* 995, demonstrated that the two internal HexNAcs were substituted at C4. Taken together this indicated a blood group A type 2 octasaccharide (GalNAcα3(Fucα2)Galβ4GlcNAcβ3 Galβ4GlcNAcβ3Galβ4Glc).

Finally, the MS^2^ spectrum of the ion at *m/z* 1436 (Fig. 8K) gave a tentative identification of a neolacto octasaccharide (Galβ4GlcNAcβ3Galβ4GlcNAcβ3Galβ4GlcNAcβ3Galβ4Glc). Here the lower mass region of the spectrum was weak, but had similarities with the spectrum in Fig. 8H. Thus, the C_4_ ion at *m/z* 747, the C_5_ ion at *m/z* 909 and the C_6_ ion at *m/z* 1112, allowed a tentative identification of an octasaccharide with Hex-HexNAc-Hex-HexNAc-Hex-HexNAc-Hex-Hex sequence, and 4-substitution of the two innermost HexNAcs was demonstrated by with the ^0,2^A_4_ fragment ion at *m/z* 646 and the ^0,2^A_6_ fragment ion at *m/z* 1011.

Thus, the oligosaccharide derived from the non-acid glycosphingolipids of GIST I had mainly type 2 core chains, with neolactotetraose (Galβ4GlcNAcβ3Galβ4Glc) as the predominant compound. There were also several other minor compounds with neolacto core chain as the Le^x^ and x_2_ pentasaccharides, the blood group A type 2 hexa- and octasaccharides, and neolacto hexa- and octasaccharides. Minor oligosaccharides with type 1 core chains (Le^b^ hexasaccharide, and blood A type 1 heptasaccharide) were also present.

The base peak chromatogram from LC-ESI/MS of the oligosaccharides obtained from GIST II also had molecular ions ranging from *m/z* 706 to *m/z* 1201 (Supplementary Fig. S3A). Here MS^2^ showed that the GIST II oligosaccharides had a globo tetrasaccharide in addition to the neolacto tetrasaccharide, a Le^a^ pentasaccharide in addition to the Le^x^ hexasaccharide, a Le^y^ hexasaccharide in addition to the Le^b^ hexasaccharide (exemplified in Supplementary Fig. S3B-G), and a blood group B heptasaccharide in addtion to the blood group A heptasaccharide (see further below). The x_2_ and blood group H type 2 pentasaccharides, the blood group A type 2 hexasaccharide, and the neolacto and blood group A type 2 octasaccharides were not detected in the GIST II sample.

A globo trisaccharide, globo and neolacto tetrasaccharides, Le^x^, x_2_ and blood group H type 2 pentasaccharides, and Le^b^ and Le^y^ hexasaccharides were characterized by LC-ESI/MS of the oligosaccharides derived from GIST III (data not shown). The glycosphingolipid-derived oligosaccharides from GIST I, GIST II and GIST III characterized by LC-ESI/MS are summarized in Table 3.

**Table 3:** Glycosphingolipid-derived oligosaccharides from the total non-acid GIST fractions identified by LC-ESI/MS.

Trivial name	Structure	GIST I	GIST II	GIST III
Neolactotetra (nLc4)	Galβ4GlcNAcβ3Galβ4Glc	+	+	+
Globotetra (Gb4)	GalNAcβ3Galα4Galβ4Glc	−	+	+
x_2_ penta (x_2_)	GalNAcβ3Galβ4GlcNAcβ3Galβ4Glc	+	−	+
H type 2 penta (H5-2)	Fucα2Galβ4GlcNAcβ3Galβ4Glc	+	−	+
Le^a^ penta (Le^a^-5)	Galβ3(Fucα4)GlcNAcβ3Galβ4Glc	−	+	−
Le^x^ penta (Le^x^-5)	Galβ4(Fucα3)GlcNAcβ3Galβ4Glc	+	+	+
Neolactohexa (nLc6)	Galβ4GlcNAcβ3Galβ4GlcNAcβ3Galβ4Glc	+	+	+
A type 2 hexa (A6-2)	GalNAcα3(Fucα2)Galβ4GlcNAcβ3Galβ4Glc	+	−	−
Le^b^ hexa (Le^b^-6)	Fucα2Galβ3(Fucα4)GlcNAcβ3Galβ4Glc	+	+	+
Le^y^ hexa (Le^y^-6)	Fucα2Galβ4(Fucα3)GlcNAcβ3Galβ4Glc	−	+	+
A type 1 hepta (A7-1)	GalNAcα3(Fucα2)Galβ(Fucα4)GlcNAcβ3Galβ4Glc	+	+	−
B type 2 hepta (B7-2)	Galα3(Fucα2)Galβ4(Fucα3)GlcNAcβ3Galβ4Glc	−	+	−
Neolactoocta (nLc8)	Galβ4GlcNAcβ3Galβ4GlcNAcβ3Galβ4GlcNAcβ3Galβ4Glc	+	−	−
A type 2 octa (A8-2)	GalNAcα3(Fucα2)Galβ4GlcNAcβ3Galβ4GlcNAcβ3Galβ4Glc	+	−	−

#### Chromatogram binding assays

In order to extend the structural information obtained by mass spectrometry the binding of carbohydrate binding ligands to the non-acid glycosphingolipid fractions of GISTI and GISTII was next examined in a chromatogram binding assay. Thereby, a distinct interaction of the Galβ4GlcNAc binding lectin from *E. cristagalli*^19^ was obtained, confirming the presence of neolactotetraosylceramide in both tumors (Fig. 2B, lanes 3 and 4). The lectin binding to the glycosphingolipids of GIST I (lane 3) was more distinct than the binding to the glycosphingolipids of GIST II (lane 4), in line with the higher amount of neolactotetraosylceramide in GIST I. The presence of glycosphingolipids with blood group A determinants in the GISTs was confirmed by the binding of anti-A antibodies (Fig. 2C, lanes 3 and 4).

### Separation of the non-acid glycosphingolipids of GIST II

After these studies the remaining material in the non-acid glycosphingolipid fraction from GIST II was separated on Iatrobeads columns, in order to enrich the slow-migrating glycosphingolipids. After pooling three glycosphingolipid-containing fractions were obtained (denoted fractions N-I to N-III).

### LC-ESI/MS of fractions N-I-N-III

The three fractions were digested by *Rhodococcus* endoglycoceramidase and the oligosaccharides obtained were analyzed by LC-ESI/MS. Thereby globotetra, neolactotetra, Le^a^ penta and Le^x^ penta saccharides were identified in fraction N-I, whereas Le^b^ hexa and Le^y^ hexa saccharides were characterized in fraction N-II (data not shown).

**Figure 9:**
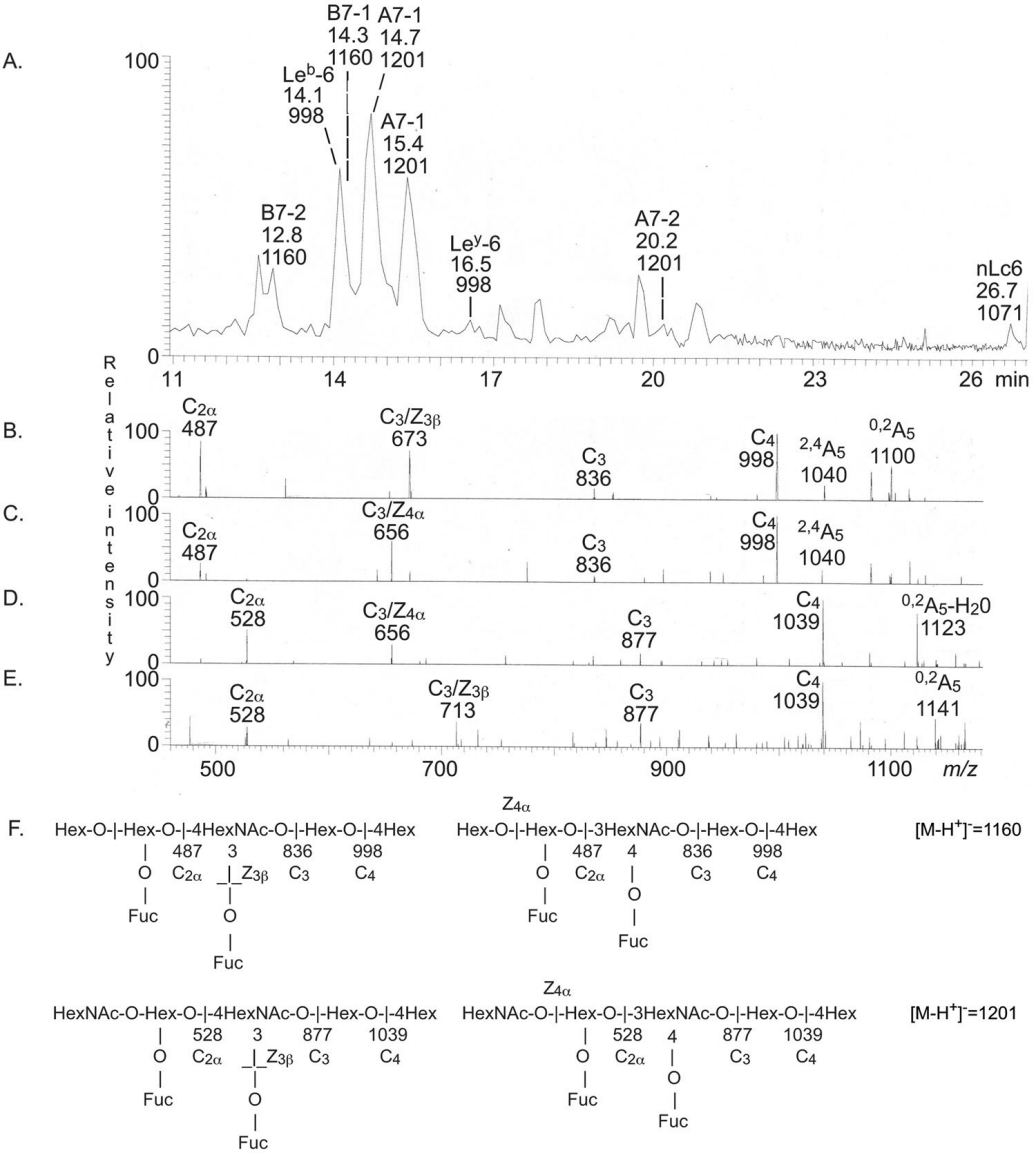
LC-ESI/MS of fraction NIII. (**A**) Base peak chromatogram from LC-ESI/MS of the oligosaccharides obtained from fraction NIII from GIST II by hydrolysis with endoglycoceramidase II from *Rhodococcus* spp. (**B**) MS^2^ of the [M − H^+^]^−^ ion at *m/z* 1160 (retention time 12.8 min). (**C**) MS^2^ of the [M − H^+^]^−^ ion at *m/z* 1160 (retention time 14.3 min). (**D**) MS^2^ of the [M − H^+^]^−^ ion at *m/z* 1201 (retention time 14.7 min). (**E**) MS^2^ of the [M − H^+^]^−^ ion at *m/z* 1201 (retention time 20.2 min). (**F**) Interpretation formulas. The identification of oligosaccharides was based on their retention times, determined molecular masses and subsequent MS^2^ sequencing. The data were processed using the Xcalibur software (version 2.0.7, Thermo Scientific, www.thermofisher.com). The oligosaccharides identified in the chromatograms were: B7-2, Galα3(Fucα2)Galβ4(Fucα3)GlcNAcβ3Galβ4Glc; Le^b^-6, Fucα2Galβ3(Fucα4)GlcNAcβ3Galβ4Glc; B7-1, Galα3(Fucα2)Galβ3(Fucα4)GlcNAcβ3Galβ4Glc; A7-1, GalNAcα3(Fucα2)Galβ3(Fucα4)GlcNAcβ3Galβ4Glc; Le^y^-6, Fucα2Galβ4(Fucα3)GlcNAcβ3Galβ4Glc; A7-2, GalNAcα3(Fucα2)Galβ4(Fucα3)GlcNAcβ3Galβ4Glc.

The base peak chromatogram from LC-ESI/MS of the oligosaccharides from fraction N-III (Fig. 9A) had two [M − H^+^]^−^ ions at *m/z* 1160, eluting at 12.8 min and 14.3 min, respectively. There were also two [M − H^+^]^−^ ions at *m/z* 1201, one major eluting at 14.7 min, and one minor eluting at 20.2 min. In addition, there were two [M − H^+^]^−^ ions at *m/z* 998, which were identified as Le^b^ and Le^y^ hexasaccharides by MS^2^ (data not shown).

A blood group B type 2/ALe^y^ heptasaccharide (Galα3(Fucα2)Galβ4(Fucα3)GlcNAcβ3Galβ4Glc) was demonstrated by MS^2^ of the [M − H^+^]^−^ ion at *m/z* 1160 at retention time 12.8 min (Fig. 9B,F). The series of C-type fragment ions (C_2_ at *m/z* 487, C_3_ at *m/z* 836, and C_4_ at *m/z* 998) established a Hex-(Fuc-)Hex-(Fuc-)HexNAc-Hex-Hex carbohydrate sequence. There was also a fragment ion at *m/z* 673, which, is caused by double glycosidic cleavage of the 3-linked branch (C_3_/Z_3_β). This ion thus demonstrated an internal 4-linked GlcNAc substituted with a Fuc at 3-position^17^.

The MS^2^ spectrum of the [M − H^+^]^−^ ion at *m/z* 1160 at retention time 14.3 min (Fig. 9C) had the same series of C-type fragment ions (C_2_ at *m/z* 487, C_3_ at *m/z* 836, and C_4_ at *m/z* 998), which thus again demonstrated a Hex-(Fuc-)Hex-(Fuc-)HexNAc-Hex-Hex sequence. The ion at *m/z* 656 is obtained by a double glycosidic cleavage of the 3-linked branch at C_3_ and Z_4_α^17^, and demonstrated an internal 3-linked GlcNAc substituted with a Fuc at C-4. Thus, here a blood group B type 1/ALe^b^ heptasaccharide (Galα3(Fucα2)Galβ3(Fucα4)GlcNAcβ3Galβ4Glc) was identified (Fig. 9F).

As above, a blood group A type 1/ALe^b^ heptasaccharide (GalNAcα3(Fucα2)Galβ3(Fucα4)GlcNAcβ3Galβ4Glc) was identified by MS^2^ of the [M − H^+^]^−^ ion at *m/z* 1201 at retention time 14.7 min (Fig. 9D,F). There was a C-type fragment ion series (C_2_ at *m/z* 528, C_3_ at *m/z* 877, and C_4_ at *m/z* 1039) identifying a HexNAc-(Fuc-)Hex-(Fuc-)HexNAc-Hex-Hex sequence (Fig. 9E), and a C_3_/Z_4_α ion at *m/z* 656, identifying an internal 3-linked GlcNAc substituted with a Fuc at C-4^17^ was also obtained. This MS^2^ spectrum was very similar to the MS^2^ spectrum of reference a blood group A type 1/ALe^b^ heptasaccharide^18^.

Finally, a blood group A type 2/ALe^y^ heptasaccharide (GalNAcα3(Fucα2)Galβ4(Fucα3)GlcNAcβ3Galβ4Glc) was characterized by MS^2^ of the [M − H^+^]^−^ ion at *m/z* 1201 at retention time 20.2 min (Fig. 9E). Again a C-type fragment ion series (C_2_ at *m/z* 528, C_3_ at *m/z* 877, and C_4_ at *m/z* 1039), identifying a HexNAc-(Fuc-)Hex-(Fuc-)HexNAc-Hex-Hex sequence (Fig. 9E), was present, and the C_3_/Z_3_β ion at *m/z* 713 identified an internal 4-linked GlcNAc substituted with a Fuc at 3-position^17^. This MS^2^ spectrum and the MS^2^ spectrum of reference blood group A type 2/ALe^y^ heptasaccharide^18^ were very similar.

Thus, fraction N-III from GIST II was a complex mixture of blood group A and B heptaosylceramides with both type 1 and type 2 core chains, and also Le^b^ and Le^y^ hexaosylceramides.

## Discussion

GISTs are the most common primary mesenchymal tumour of the human alimentary tract, counting for less than 1% of all gastrointestinal tumours and about 5% all sarcomas. GISTs originate from the interstitial cells of Cajal in the muscularis propria of the gastrointestinal tract. These cells are specialized smooth muscle cells, which receive inputs from nerve cells within the myenteric and submucosal plexuses, and also communicate electrically with smooth muscle cells in the longitudinal and circular muscle layers^20,21^, and thereby contribute to peristalsis which facilitates propulsion of intestinal contents, and small intestine segmentation which facilitates absorption of nutrients^22^.

The interstitial cells of Cajal and the intestinal circular smooth muscle cells both originate from a common, mesodermally derived mesenchymal pluripotential precursor^22,23^. These mesenchymal progenitors express both the receptor tyrosine kinase c-Kit and smooth muscle myosin heavy chain. Cells that will differentiate into interstitial cells of Cajal maintain the expression of c-Kit, while the cells destined to become smooth muscle cells up‐regulate the synthesis of myofilament proteins and down-regulate Kit^24,25^.

It is obvious that the glycosphingolipids of the Cajal cells can not be isolated for structural characterization, and studies of glycosphingolipids of human smooth mucle are rare. However, by binding studies using a battery of monoclonal antibodies, Gillard et al.^26^ found that the major non-acid glycosphingolipids of cultured smooth muscle cells, derived from human umbilical cord veins, were lactosylceramide, globotri- and globotetraosylceramide. There were also small amounts of neolactotetraosylceramide, Le^x^ pentaosylceramide and globopentaosylceramide/SSEA-3 (Galβ3GalNAcβ3Galα4Galβ4Glcβ1Cer). GM3 and Neu5Acα3-neolactotetraosylceramide were the major gangliosides, whereas no GD3 ganglioside was detected. Most of the long chain gangliosides had a neolacto core (as *e.g*. Neu5Acα3-dimeric-Le^x^ nonaosylceramide (Neu5Acα3Galβ4(Fucα4)GlcNAcβ3Galβ4(Fucα4)GlcNAcβ3Galβ4Glcβ1Cer)), or globo core (as *e.g*. Neu5Acα3-globopentaosylceramide/SSEA-4 (Neu5Acα3Galβ3GalNAcβ3Galα4Galβ4Glcβ1Cer)). There was no binding of a monoclonal anti-H type 2 antibody to the smooth muscle cell glycosphingolipids, and binding anti-A or anti-B antibodies was not tested.

In this study, the non-acid and acid glycosphingolipids of three GIST tumours were characterised with the combination of thin-layer chromatography, binding of carbohydrate recognizing ligands and mass spectrometry. The acid fractions of GIST had sulfatide, and GM3 was the major ganglioside. In contrast to the previous study of smoth muscle cell glycosphingolipids, GD3 and the several gangliosides with ganglio core chain (GM1, GD1a, GT1b and GQ1b) were characterized. In the non-acid fractions of the tumours globotetraosylceramide, neolactotetraosylceramide, the x_2_ pentaosylceramide, and glycosphingolipids with terminal blood group A, B, H, Le^x^, Le^a^, Le^b^ and Le^y^ determinants were characterized. The expression of blood group A, B, and H glycosphingolipids was in agreement with the ABO phenotype of the patients, and the relative amounts of the glycosphingolipids with blood group determinants were different in the three tumour samples. Most likely this is a reflection of the blood group determinants expressed by the cells of origin, *i.e*. the interstitial cells of Cajal.

Overall the glycosphingolipid patterns of the three gastric GISTs had a high similarity with the glycosphingolipids of the human stomach^18,27^. The non-acid glycosphingolipids of human stomach had a high degree of structural complexity, and the composition of glycosphingolipids differed for individuals of separate blood groups. The major complex glycosphingolipids of blood group O(Rh-)P stomach were neolactotetraosylceramide, the Le^x^, Le^a^, and H type 2 pentaosylceramides, and the Le^y^ hexaosylceramide. The A(Rh +)P, and A(Rh +)p stomachs had lactotetraosylceramide, the x_2_ and the H type 1 pentaosylceramides, neolactohexaosylceramide the Le^b^ hexaosylceramide and the A type 1/ALe^b^ heptaosylceramide^18^. The acid human stomach glycosphingolipids characterized were sulfatide and the gangliosides GM3, GD3, GM1, Neu5Acα3-neolactotetraosylceramide, Neu5Acα3-neolactohexaosylceramide and Neu5Acα3-neolactooctaosylceramide, GD1a and GD1b^27^. The major difference observed was the relatively low levels of sulfatide in the GIST tumours compared to the human stomach^27^.

Several gangliosides are overexpressed in cancers, and a substantial number of cancer immunotherapies targeting such gangliosides have been developed^28,29^. Already in 1985, a phase I clinical trial with an anti-GD3 antibody demonstrated regression of tumours, activation of T-cells, antibody-dependant-cell-cytotoxicity, and complement dependent cytotoxicity in malignant melanoma patients^30^. More recently an anti-GD2 monoclonal antibody Dinutuximab (Unituxin) has been approved by the Food Drug Administration (FDA) and European Medicines Agency (EMA) for the treatment of high-risk neuroblastomas^31^. Dinutuximab is used in combination with interleukin-2, granulocyte–macrophage colony-stimulating factor, and isotretinoin (13-*cis-*retinoic acid) for maintenance treatment of pediatric patients with high-risk neuroblastoma who has achieved at least a partial response to first-line multiagent, multimodality therapy^32^. Dinutuximab gave an increased the 2-year event-free survival and overall survival when compared to standard treatment in phase III trials^33^.

This study is the first structural characterization of GIST glycosphingolipids, and demonstrates the presence of glycosphingolipids with blood group ABO and Lewis determinants, along with several complex gangliosides, in these tumors derived from the interstitial cells of Cajal, which are specialized smooth muscle cells. Further studies will be needed to clarify the potential value of these findings for tumour biology, response to therapy and patient survival.

## Methods

### Glycosphingolipids preparations

The study was conducted according to the tenets of the Declaration of Helsinki, and was approved by the Regional Ethics Committee of Gothenburg (No. 621-18 (decision 2018-09-19)). All patients provided written informed consents before enrollment in the study.

Three gastric GIST tumours (denoted GIST I, GIST II and GIST III) were collected at the Sahlgrenska University Hospital. The tumours were kept at − 70 °C. The isolation of total acid and total non-acid glycosphingolipids was done by the method described by Karlsson^14^. The tumours were lyophilized, followed by Soxhlet extraction with mixtures of chloroform and methanol (2:1 and 1:9, by volume). The resulting material was pooled and subjected to mild alkaline hydrolysis followed by dialysis. Thereafter non-polar compounds were removed by chromatography on a silicic acid column. Acid and non-acid glycosphingolipids were separated by ion change chromatography on a DEAE-cellulose column. In order to separate the non-acid glycosphingolipids from alkali-stable phospholipids, the non-acid fractions were then acetylated and separated on a second silicic acid column, followed by deacetylation and dialysis. Final purifications are performed by chromatography on DEAE-cellulose and silicic acid columns.

After the first characterization by binding assays and LC-ESI/MS, the acid glycosphingolipids from GIST II were separated on an Iatrobeads column eluted with increasing volumes of methanol in chloroform. The fractions obtained were analyzed by thin-layer chromatography and anisaldehyde/resorcinol detection, and thereafter pooled according to their mobility on thin-layer chromatograms, resulting in five subfractions, which were denoted fractions A-1 to A-5.

The non-acid glycosphingolipids from GIST II were separated on an Iatrobeads column eluted with eluted with chloroform:methanol:water (60:35:8, by volume), 29 × 1 ml. The fractions were analysed by thin-layer chromatography, and after pooling according to their mobility on thin-layer chromatograms, three glycosphingolipid-containing fractions were obtained (denoted fractions N-I to N-III).

### Reference glycosphingolipids

Total acid and non-acid glycosphingolipid fractions were isolated as described^14^. Individual glycosphingolipids were isolated by repeated chromatography on silicic acid columns and by HPLC, and identified by mass spectrometry^16,34^ and ^1^H-NMR spectroscopy^35^.

### Thin-layer chromatography

Thin-layer chromatography was performed on aluminium- or glass-backed silica gel 60 high-performance thin-layer plates (Merck, Germany). Glycosphingolipid mixtures (40 μg), or pure glycosphingolipids (4 μg), were applied to the plates, and eluted with chloroform/methanol/water 60:35:8 (by volume). Chemical detection was done with anisaldehyde^36^ or resorcinol^37^.

### Chromatogram binding assays

The carbohydrate binding ligands and dilutions used in the chromatogram binding assays are given in Table 4. Binding of antibodies to glycosphingolipids separated on thin-layer chromatograms was performed as described by Barone et al.^38^. After elution, the dried thin-layer chromatograms plates were treated with a mixture of 0.5% polyisobutylmethacrylate (w/v) in diethylether/n-hexane (1:5 v/v) for 1 min, and then air-dried. Thereafter followed a 2 h incubation at room temperature with PBS (phosphate-buffered saline, pH 7.3) containing 2% (w/v) bovine serum albumin, 0.1% (w/v) NaN_3_ and 0.1% (w/v) Tween 20 (BSA/PBS/TWEEN) to reduce unspecific binding. Then, the chromatograms were incubated for 2 h at room temperature with suspensions of monoclonal antibodies diluted in BSA/PBS/TWEEN. The chromatograms were washed with PBS, and the chromatograms were thereafter incubated for 2 h with ^125^I-labeled (labeled by the IODO-GEN method according to the manufacturer’s instructions (Pierce/Thermo Scientific)) rabbit anti-mouse antibodies (Pierce/Thermo Scientific) diluted in BSA/PBS/TWEEN. Finally, the chromatograms were washed with PBS, dried and autoradiographed overnight using x-ray films (Carestream; 8,941,114).

**Table 4:** Carbohydrate binding ligands used in chromatogram binding assays.

Ligand	Clone	Manufacturer	Dilution	Isotype
*E. cristagalli* lectin	–	Vector Labs	1:100	–
Cholera toxin B-subunits	–	List Labs	1:100	–
Anti-GD3	MB3.6	BD Biosciences	1:100	IgG3
Anti-GD1a	GD1a-1	Millipore	1:100	IgG1
Anti-Neu5Acα3-neolacto	LM1:1a	Ref.^41^	1:100	IgM
Anti-Neu5Acα6-neolacto	LM4:2	Ref.^42^	1:100	IgG
Anti-A	HE-195	Sigma-Aldrich	1:500	IgM

Binding of ^125^I-labeled *Erythrina cristagalli* lectin (Sigma-Aldrich), and cholera toxin B-subunits (List Laboratories), to glycosphingolipids on thin-layer chromatograms was done as described^19,39^.

### LC-ESI/MS of native glycosphingolipids

The native glycosphingolipid fractions were analyzed by LC-ESI/MS as described^18,40^. Aliquots of the glycosphingolipid fractions were dissolved in methanol:acetonitrile 75:25 (by volume) and separated on a 200 × 0.250 mm column, packed in-house with 5 µm polyamine II particles (YMC Europe GmbH, Dinslaken, Germany). An autosampler, HTC-PAL (CTC Analytics AG, Zwingen, Switzerland) equipped with a cheminert valve (0.25 mm bore) and a 2 µl loop, was used for sample injection. An Agilent 1100 binary pump (Agilent Technologies, Palo Alto, CA) delivered a flow of 250 µl/min, which was splitted down in an 1/16″ microvolume-T (0.15 mm bore) (Vici AG International, Schenkon, Switzerland) by a 50 cm × 50 µm i.d. fused silica capillary before the injector of the autosampler, allowing approximately 2–3 µl/min through the column. Samples were eluted with an aqueous gradient (A:100% acetonitrile to B: 10 mM ammonium bicarbonate). The gradient (0–50% B) was eluted for 40 min, followed by a wash step with 100% B, and equilibration of the column for 20 min. The samples were analyzed in negative ion mode on a LTQ linear quadrupole ion trap mass spectrometer (Thermo Electron, San José, CA), with an IonMax standard ESI source equipped with a stainless steel needle kept at − 3.5 kV. Compressed air was used as nebulizer gas. The heated capillary was kept at 270 °C, and the capillary voltage was − 50 kV. Full scan (*m/z* 500–1800, two microscans, maximum 100 ms, target value of 30,000) was performed, followed by data-dependent MS^2^ scans (two microscans, maximun 100 ms, target value of 10.000) with normalized collision energy of 35%, isolation window of 2.5 units, activation q = 0.25 and activation time 30 ms). The threshold for MS^2^ was set to 500 counts.

Data acquisition and processing were conducted with Xcalibur software version 2.0.7 (Thermo Scientific, Waltham, MA). Manual assignment of glycosphingolipid sequences was done with the assistance of the Glycoworkbench tool (Version 2.1), and by comparison of retention times and MS^2^ spectra of reference glycosphingolipids.

### Endoglycoceramidase digestion and LC-ESI/MS

The non-acid glycosphingolipids were digested with endoglycoceraminidase and the oligosaccharides thereby obtained were analyzed by LC-ESI/MS^16,18,40^. The glycosphingolipids (50 µg) were resuspended in 100 µl 0.05 M sodium acetate buffer, pH 5.0, containing 120 µg sodium cholate, and sonicated briefly. Thereafter, 1 mU of endoglycoceramidase II from *Rhodococcus* spp. (Takara Bio Europe S.A., Gennevilliers, France) was added, and the mixture was incubated at 37 °C for 48 h. The reaction was stopped by addition of chloroform/methanol/water to the final proportions 8:4:3 (by volume). The oligosaccharide-containing upper phase thus obtained was separated from detergent on a Sep-Pak QMA cartridge (Waters). The eluant containing the oligosaccharides was dried under nitrogen and under vacuum.

The glycosphingolipid-derived oligosaccharides were resuspended in 50 µl water and analyzed by LC-ESI/MS as described^16,18,40^. The oligosaccharides were separated on a column (100 × 0.250 mm) packed in-house with 5 µm porous graphite particles (Hypercarb, Thermo-Hypersil, Runcorn, UK). An autosampler, HTC-PAL (CTC Analytics AG, Zwingen, Switzerland) equipped with a cheminert valve (0.25 mm bore) and a 2 µl loop, was used for sample injection. An Agilent 1100 binary pump (Agilent technologies, Palo Alto, CA) delivered a flow of 250 µl/min, which was split down in an 1/16″ microvolume-T (0.15 mm bore) (Vici AG International, Schenkon, Switzerland) by a 50 cm × 50 µm i.d. fused silica capillary before the injector of the autosampler, allowing approximately 3–5 µl/min through the column. The oligosaccharides (3 µl) were injected on to the column and eluted with an acetonitrile gradient (A: 10 mM ammonium bicarbonate; B: 10 mM ammonium bicarbonate in 80% acetonitrile). The gradient (0–45% B) was eluted for 46 min, followed by a wash step with 100% B, and equilibration of the column for 24 min. A 30 cm × 50 µm i.d. fused silica capillary was used as transfer line to the ion source.

The oligosaccharides were analyzed in negative ion mode on an LTQ linear quadrupole ion trap mass spectrometer (Thermo Electron, San José, CA). The IonMax standard ESI source on the LTQ mass spectrometer was equipped with a stainless steel needle kept at − 3.5 kV. Compressed air was used as nebulizer gas. The heated capillary was kept at 270 °C, and the capillary voltage was − 50 kV. Full scan (*m/z* 380-2 000, 2 microscans, maximum 100 ms, target value of 30 000) was performed, followed by data dependent MS^2^ scans of the three most abundant ions in each scan (2 microscans, maximum 100 ms, target value of 10 000). The threshold for MS^2^ was set to 500 counts. Normalized collision energy was 35%, and an isolation window of 3 u, an activation q = 0.25, and an activation time of 30 ms, was used. Selected fractions were also analyzed at *m/z* 1300–2000. Data acquisition and processing were conducted with version 2.0.7 (Thermo Scientific, Waltham, MA).

Manual assignment of glycan sequences was done on the basis of knowledge of mammalian biosynthetic pathways, with the assistance of the Glycoworkbench tool (Version 2.1), and by comparison of retention times and MS^2^ spectra of oligosaccharides from reference glycosphingolipids^16^.

## Supplementary information

Supplementary Information 1.

## Abbreviations

GIST: Gastrointestinal stromal tumours
LC-ESI/MS: Liquid chromatography electrospray ionization mass spectrometry

## References

[1] Rosai, J. Gastrointestinal tract: Esophagus–stomach–small bowel–appendix–large bowel–anus in *Rosai and Ackerman´s Surgical Pathology*, 10 ed., 1660–1773 (Philadelphia: Elsevier - Health Sciences Division, 2011).

[2] Nilsson B (2005). Gastrointestinal stromal tumors: the incidence, prevalence, clinical course, and prognostication in the preimatinib mesylate era—a population-based study in western Sweden. Cancer.

[3] Parab TM (2019). Gastrointestinal stromal tumors: a comprehensive review. J. Gastrointest. Oncol..

[4] Wada R, Arai H, Kure S, Peng WX, Naito Z (2016). Wild type GIST: clinicopathological features and clinical practise. Pathol. Int..

[5] Andersson J (2006). Gastrointestinal stromal tumors with KIT exon 11 deletions are associated with poor prognosis. Gastroenterology.

[6] Özer-Stillman I, Strand L, Chang J, Mohamed AF, Tranbarger-Freier KE (2015). Meta-analysis for the association between overall survival and progression-free survival in gastrointestinal stromal tumor. Clin. Cancer Res..

[7] Mantese G (2019). Gastrointestinal stromal tumor: epidemiology, diagnosis, and treatment. Curr. Opin. Gastroenterol..

[8] Fuster MM, Esko JD (2005). The sweet and sour of cancer: glycans as novel therapeutic targets. Nat. Rev. Cancer..

[9] Varki, A., Kannagi, R. & Toole, B. P. 2009. Glycan changes in cancer in *Essentials of Glycobiology* (eds. Vark,i A., Cummings, R.D., Esko, J.D., Freeze, H.H., Stanley, P., Bertozzi, C.R., Hart, G.W. & Etzler, M.E.) 2nd ed., 617–632 (New York: Cold Spring Harbor Laboratory Press, 2009). 20301239

[10] Pearce OMT (2018). Cancer glycan epitopes: biosynthesis, structure and function. Glycobiology.

[11] Terzi H (2015). New method: are tumor markers in vaginal-washing fluid significant in the diagnosis of primary ovarian carcinoma?. Eur. J. Gynaecol. Oncol..

[12] Kim J-H, Jun K-H, Jung H, Park I-S, Chin H-M (2014). Prognostic value of preoperative serum levels of five tumor markers (carcinoembryonic antigen, CA19-9, alpha-fetoprotein, CA72-4, and CA125) in gastric cancer. Hepatogastroenterology.

[13] Zhang D, Yu M, Xu T, Xiong B (2013). Predictive value of serum CEA, CA19-9 and CA125 in diagnosis of colorectal liver metastasis in Chinese population. Hepatogastroenterology.

[14] Karlsson K-A (1987). Preparation of total non-acid glycolipids for overlay analysis of receptors for bacteria and viruses and for other studies. Methods Enzymol..

[15] Hsu FF, Turk J (2004). Studies on sulfatides by quadrupole ion-trap mass spectrometry with electrospray ionization: structural characterization and the fragmentation processes that include an unusual internal galactose residue loss and the classical charge-remote fragmentation. J. Am. Soc. Mass Spectrom..

[16] Karlsson H, Halim A, Teneberg S (2010). Differentiation of glycosphingolipid-derived glycan structural isomers by liquid chromatography–mass spectrometry. Glycobiology.

[17] Chai W, Piskarev V, Lawson AM (2001). Negative-ion electrospray mass spectrometry of neutral underivatized oligosaccharides. Anal. Chem..

[18] Jin C, Barone A, Borén T, Teneberg S (2018). *Helicobacter pylori* binding non-acid glycosphingolipids in the human stomach. J. Biol. Chem..

[19] Teneberg S, Jovall P-Å, Ångström J, Karlsson K-A (1994). Characterization of binding of Galβ4GlcNAc-specific lectins from *Erythrina christagalli* and *Erythrina corallodendron* to glycosphingolipids. detection, isolation and characteriztion of a novel glycosphingolipid of bovine buttermilk. J. Biol. Chem..

[20] Feher, J. Intestinal and Colonic Motility in *Quantitative human physiology*. 711–720 (Amsterdam: Academic Press, 2012)

[21] Sanders KM, Koh SD, Ro S, Ward SM (2012). Regulation of gastrointestinal motility–insights from smooth muscle biology. Nat. Rev. Gastroenterol. Hepatol..

[22] Huizinga JD (2014). The origin of segmentation motor activity in the intestine. Nat. Commun..

[23] Sanders KM, Ward SM, Koh SD (2014). Interstitial cells: regulators of smooth muscle function. Physiol. Rev..

[24] Kluppel M, Huizinga JD, Malysz J, Bernstein A (1998). Developmental origin and kit-dependent development of the interstitial cells of cajal in the mammalian small intestine. Dev. Dyn..

[25] Sanders KM, Ordog T, Koh SD, Torihashi S, Ward SM (1999). Development and plasticity of interstitial cells of Cajal. Neurogastroenterol. Motil..

[26] Gillard BK, Jones MA, Marcus DM (1987). Glycosphingolipids of human umbilical vein endothelial cells and smooth muscle cells. Arch. Biochem. Biophys..

[27] Benktander J, Barone A, Madar Johansson M, Teneberg S (2018). *Helicobacter pylori* SabA binding gangliosides of human stomach. Virulence.

[28] Danotti DL, Vilcaes AA, Demichelis VT, Ruggiero FM, Rodriguez-Walker M (2013). Glycosylation of glycolipids in cancer basis for development of novel therapeutic approaches. Front. Oncol..

[29] Groux-Degroote S, Guérardel Y, Delannoy P (2017). Gangliosides: structures, biosynthesis, analysis and roles in cancer. ChemBioChem.

[30] Houghton AN (1985). Mouse monoclonal IgG3 antibody detecting GD3 ganglioside: a phase I trial in patients with malignant melanoma. Proc. Natl. Acad. Sci. USA.

[31] Dhillon S (2015). Dinutuximab: first global approval. Drugs.

[32] Keyel ME, Reynolds CP (2019). Spotlight on dinutuximab in the treatment of high-risk neuroblastoma: development and place in therapy. Biologics.

[33] Peinemann F, van Dalen EC, Enk H, Tytgat GA (2019). Anti-GD2 antibody-containing immunotherapy postconsolidation therapy for people with high-risk neuroblastoma treated with autologous haematopoietic stem cell transplantation. Cochrane Database Syst. Rev.

[34] Samuelsson BE, Pimlott W, Karlsson K-A (1990). Mass spectrometry of mixtures of intact glycosphingolipids. Methods Enzymol..

[35] Koerner TAW, Prestegard JH, Demou PC, Yu RK (1983). High-resolution proton NMR studies of gangliosides. 1. Use of homonuclear spin-echo J-correlated spectroscopy for determination of residue composition and anomeric configurations. Biochemistry.

[36] Waldi, D. Sprühreagentien für die dünnschicht-chromatographie in *Dünnschicht-Chromatographie* (ed. Stahl, E.) 496–515 (Berlin: Springer-Verlag, 1962).

[37] Svennerholm L, Fredman P (1980). A procedure for the quantitative isolation of brain gangliosides. Biochim. Biophys. Acta.

[38] Barone A (2014). Sialyl-lactotetra: a novel cell surface marker of undifferentiated human pluripotent stem cells. J. Biol. Chem..

[39] Ångström J, Teneberg S, Karlsson K-A (1994). Delineation and comparison of ganglioside binding epitopes for the toxins from *Vibrio cholerae*, *Escherichia coli* and *Clostridium tetani*. Proc. Natl. Acad. Sci. USA.

[40] Johansson MM (2015). Characterization of moose intestinal glycosphingolipids. Glycoconj. J..

[41] Svennerholm L (1991). Gangliosides in human fetal brain. J. Neurochem..

[42] Nilsson O, Lindholm L, Holmgren J, Svennerholm L (1985). Monoclonal antibodies raised against NeuAcα2-6neolactotetraosylceramide detect carcinomaassociated gangliosides. Biochim. Biophys. Acta.

