# Supplementary material for: Characterization of glycosphingolipids from gastrointestinal stromal tumours
Source: Sci Rep. 2020 Nov 9;10:19371. doi: 10.1038/s41598-020-76104-3 (PMC7653041; doi:10.1038/s41598-020-76104-3)
Supplement: Supplementary file 1 — Supplementary Information 1. [file 41598_2020_76104_MOESM1_ESM.pdf]

Supplementary information

Characterization of Glycosphingolipids from Gastrointestinal Stromal Tumours

Licínia Santos, Chunsheng Jin, Taťána Gazárková, Anders Thornell, Olov Norlén, Karin Säljö & Susann Teneberg

Figures S1-S3

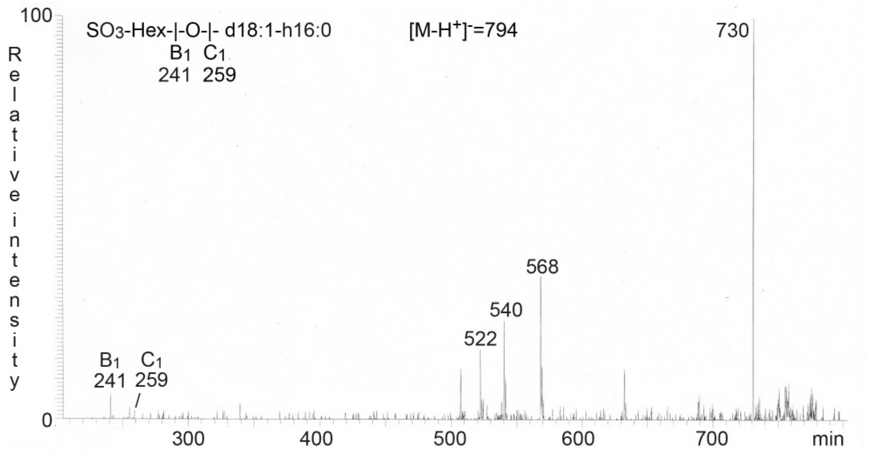

Figure S1. Characterization of the total acid glycosphingolipid fraction from GIST II by LC-ESI/MS. MS^2 of the $[M\text{-}H^+]^-$ ion at *m/z* 794 (retention time 8.2 min) identified sulfatide (SO_3-3Galβ1Cer) with d18:1-h16:0 ceramide. The MS^2 spectrum had a B_1 ion at *m/z* 241, and a C_1 ion at *m/z* 259, demonstrating a terminal SO_3-Hex. The ions at *m/z* 522, 540 and 568 were due to loss of the fatty acyl from the molecular ion.

B. *m/z* 706	Hex-O-4HexNAc-O-|-Hex-O-|-4Hex<br>382 544<br>C_2 C_3	Neolactotetra
C. *m/z* 852<br>RT 19.5	Hex-O-4HexNAc-O-|-Hex-O-|-4Hex<br>_|_ Z3β 528 690<br>O C_2 C_3<br>| Fuc	Le^x penta
D. *m/z* 852<br>RT 26.0	Fuc-O-Hex-O-|-4HexNAc-O-|-Hex-O-|-4Hex<br>325 528 690<br>C_2 C_3 C_4	H type 2 penta
E. *m/z* 909	HexNAc-O-Hex-O-|-4HexNAc-O-|-Hex-O-|-4Hex<br>382 585 747<br>C_2 C_3 C_4	x_2 penta
F. *m/z* 998	Z3α<br>Fuc-O-Hex-|-O-3HexNAc-O-|-Hex-O-|-4Hex<br>| 674 836<br>O C_3 C_4<br>| Fuc	Le^b hexa
G. *m/z* 1071	Hex-O-4HexNAc-O-|-Hex-O-|-4HexNAc-O-|-Hex-O-|-4Hex<br>382 544 747 909<br>C_2 C_3 C_4 C_5	Neolactohexa
H. *m/z* 1055	HexNAc-O-Hex-O-|-4HexNAc-O-|-Hex-O-|-4Hex<br>| 528 731 893<br>O C_2 C_3 C_4<br>| Fuc	A type 2 hexa
I. *m/z* 1201	$Z_{4\alpha}$<br>HexNAc-O-|-Hex-O-|-3HexNAc-O-|-Hex-O-|-4Hex<br>| 528 4 877 1039<br>O $C_{2\alpha}$ | C_3 C_4<br>| O<br>Fuc | Fuc	A type 1 hepta

Figure S2. Interpretation formulas for Figure 8.

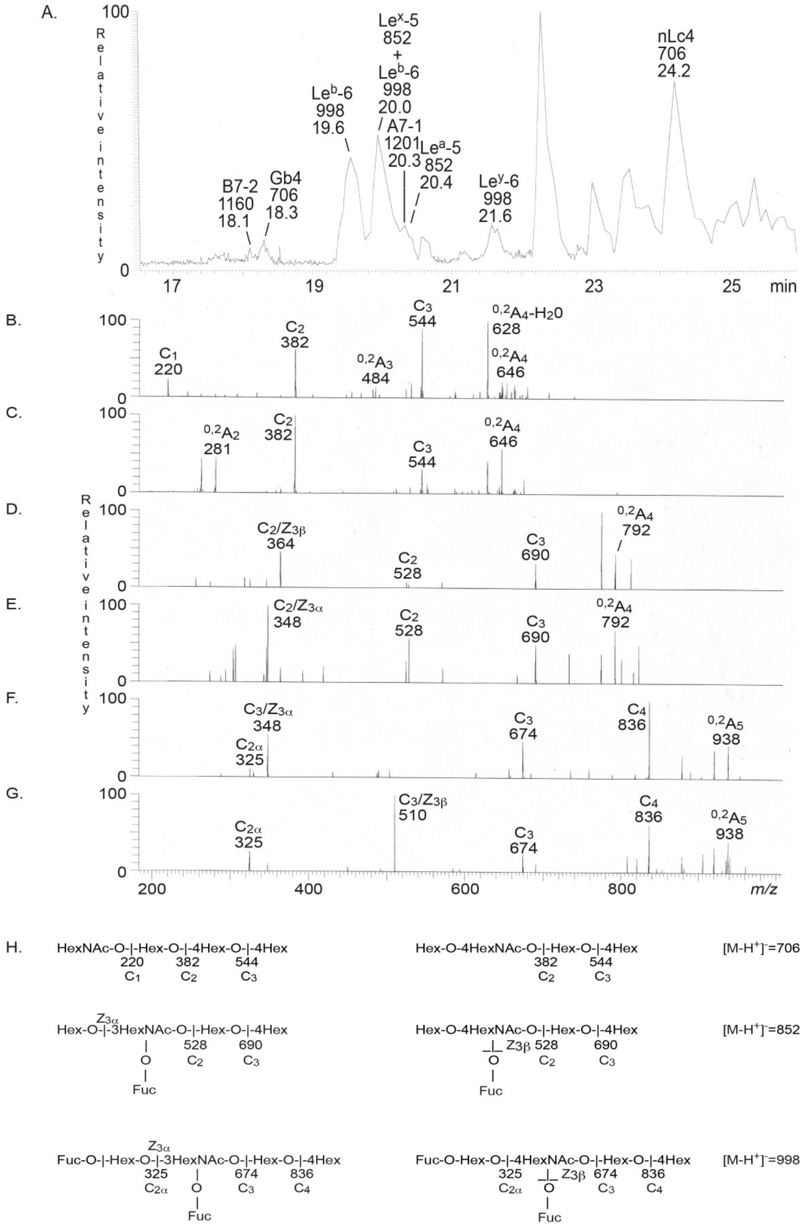

A.
Relative intensity
100
0
B7-2 1160 18.1
Gb4 706 18.3
Leb-6 998 19.6
Lex-5 852 + Leb-6 998 20.0
A7-1 1201 20.3
Lea-5 852 20.4
Ley-6 998 21.6
nLc4 706 24.2
17 19 21 23 25 min
B.
C1 220
C2 382
0,2A3 484
C3 544
0,2A4-H20 628
0,2A4 646
C.
0,2A2 281
C2 382
C3 544
0,2A4 646
D.
C2/Z3β 364
C2 528
C3 690
0,2A4 792
E.
C2/Z3α 348
C2 528
C3 690
0,2A4 792
F.
C3/Z3α 348
C2α 325
C3 674
C4 836
0,2A5 938
G.
C3/Z3β 510
C2α 325
C3 674
C4 836
0,2A5 938
200 400 600 800 m/z
H.
HexNAc-O-|-Hex-O-|-4Hex-O-|-4Hex
220 382 544
C1 C2 C3
Hex-O-4HexNAc-O-|-Hex-O-|-4Hex
382 544
C2 C3
[M-H+]-=706
Z3α
Hex-O-|-3HexNAc-O-|-Hex-O-|-4Hex
528 690
C2 C3
O
Fuc
Hex-O-4HexNAc-O-|-Hex-O-|-4Hex
Z3β 528 690
C2 C3
O
Fuc
[M-H+]-=852
Z3α
Fuc-O-|-Hex-O-|-3HexNAc-O-|-Hex-O-|-4Hex
325 674 836
C2α C3 C4
O
Fuc
Fuc-O-Hex-O-|-4HexNAc-O-|-Hex-O-|-4Hex
325 Z3β 674 836
C2α C3 C4
O
Fuc
[M-H+]-=998

Figure S3. Characterization of the non-acid glycosphingolipids of GIST II. LC-ESI/MS of the oligosaccharides obtained from the total non-acid glycosphingolipid fractions from GIST II by hydrolysis with endoglycoceramidase II from *Rhodococcus* spp.
(A) Base peak chromatogram from LC-ESI/MS of the oligosaccharides obtained from the total non-acid glycosphingolipid fractions from GIST II by hydrolysis with endoglycoceramidase II from *Rhodococcus* spp.
(B) MS^2 of the $[M-H^+]^-$ ion at *m/z* 706 (retention time 18.3 min). The C-type fragment ion series (C_1 at *m/z* 220, C_2 at *m/z* 382, and C_3 at *m/z* 544), demonstrated a HexNAc-Hex-Hex-Hex sequence, and the $^{0,2}A_3$ fragment ion at *m/z* 484 demonstrated a 4-substituted internal Hex. Taken together this identified a globotetra saccharide (GalNAcβ3Galα4Galβ4Glc).
(C) MS^2 of the $[M-H^+]^-$ ion at *m/z* 706 (retention time 24.2 min). The C-type fragment ion series (C_2 at *m/z* 382 and C_3 at *m/z* 544), demonstrated a Hex-HexNAc-Hex-Hex sequence, while the $^{0,2}A_2$ ion at *m/z* 281 demonstrated 4-substitution of the HexNAc. Thus, a neolactotetra saccharide (Galβ4GlcNAcβ3Galβ4Glc) was tentatively identified.
(D) MS^2 of the $[M-H^+]^-$ ion at *m/z* 852 (retention time 20.0 min). The ion at *m/z* 364 is obtained by double glycosidic cleavage of the 3-linked branch at C_3 and $Z_{3\beta}$, and characteristic for an internal 4-linked GlcNAc substituted with a Fuc at 3-position (Chai et al. 2001). Taken together with the C_2 ion at *m/z* 528 and the C_3 ion at *m/z* 690, this identified a Le^x pentasaccharide (Galβ4(Fucα3)GlcNAcβ3Galβ4Glc).
(E) MS^2 of the $[M-H^+]^-$ ion at *m/z* 852 (retention time 20.4 min). The MS^2 spectrum had a prominent fragment ion at *m/z* 348. This type of ion is diagnostic for an internal 3-linked GlcNAc substituted with a Fuc at C-4 [2], and is a double glycosidic cleavage of the 3-linked branch at C_3 and $Z_{3\alpha}$. Taken together with the C_2 ion at *m/z* 528 and the C_3 ion at *m/z* 690, this demonstrated a Le^a pentasaccharide (Galβ3(Fucα4)GlcNAcβ3-Galβ4Glc).
(F) MS^2 of the $[M-H^+]^-$ ion at *m/z* 998 (retention time 19.6 min). The MS^2 spectrum also had a prominent fragment ion at *m/z* 348, demonstrating for an internal 3-linked GlcNAc substituted with a Fuc at C-4 [2]. C-type fragment ions were present at *m/z* 674 (C_3) and *m/z* 836 (C_4), and taken together this indicated a Le^b hexasaccharide (Fucα2Galβ3(Fucα4)GlcNAcβ3Galβ4Glc).
(G) MS^2 of the $[M-H^+]^-$ ion at *m/z* 998 (retention time 21.5 min). The ion at *m/z* 510 is obtained by double glycosidic cleavage of the 3-linked branch at C_3 and $Z_{3\beta}$, and characteristic for an internal 4-linked GlcNAc substituted with a Fuc at 3-position [2]. Taken together with the $C_{2\alpha}$ ion at *m/z* 325 and the C_4 ion at *m/z* 836, a Le^y hexasaccharide (Fucα2Galβ4(Fucα3)GlcNAcβ3Galβ4Glc) was thus identified.
(H) Interpretation formulas.

References

1. Hsu, F.F. & Turk, J. Studies on sulfatides by quadrupole ion-trap mass spectrometry with electrospray ionization: Structural characterization and the fragmentation processes that include an unusual internal galactose residue loss and the classical charge-remote fragmentation. *J. Am. Soc. Mass Spectrom*. **15,** 536-546 (2004).

2. Chai, W., Piskarev, V. & Lawson, A.M. Negative-ion electrospray mass spectrometry of neutral underivatized oligosaccharides. *Anal .Chem*. **73,** 651-657 (2001).
